# How to assess? Student preferences for methods to assess experiential learning: A best-worst scaling approach

**DOI:** 10.1371/journal.pone.0276745

**Published:** 2022-10-27

**Authors:** Grace Melo, Diego Monteza, Greg Colson, Yu Yvette Zhang

**Affiliations:** 1 Department of Agricultural Economics, Texas A&M University, College Station, Texas, United States of America; 2 Departamento de Economia Agraria, Pontificia Universidad Catolica de Chile, Santiago, Chile; 3 Department of Agricultural and Applied Economics, University of Georgia, Athens, Georgia, United States of America; The University of Sydney, AUSTRALIA

## Abstract

Transitioning from traditional in-person classroom formats to online instructional delivery methods and online student assessments during the COVID-19 pandemic was a significant challenge to effective teaching, learning, and evaluation. Although there is a growing literature assessing the relative efficacy of different online teaching techniques, previous literature has not analyzed, from the student perspective, what methods are preferred for evaluating performance in experiential learning courses. How students perceive assessment methods is critical because it can affect their learning experience and academic achievements. To better understand student preferences for assessment methods, the best-worst scaling approach was used in two online surveys of 218 undergraduate students enrolled in experiential learning-based programs during the COVID-19 pandemic. Analysis of student responses indicates students’ highest levels of support for assessments that emphasize the development of critical thinking skills and professional skills, such as case studies. Most students would prefer assessments that are driving (develop different skills such as creative thinking) and realistic (develop skills transferable to the real world), while only a few (< 1%) prefer assessments that are fast (involve little time), frequent, safe (has preventive measures to eliminate cheating), or strategic (high probability of getting good grades).

## Introduction

A variety of pedagogical methodologies have been developed in higher education to improve student learning and to provide students with a wide range of skills necessary to meet recent work demands (e.g., communication, hands-on experience, etc.) [1–4]. School closures and disruptions due to the COVID-19 pandemic resulted in the development of a mixture of pedagogical methods (e.g., fully online, hybrid, and face-to-face) and innovative evaluation approaches [5]. As a result, a growing literature has emerged exploring experiential learning innovations suitable for academic programs.

Instructors adopting pedagogical innovations have faced several challenges that can prevent their students from achieving the expected learning outcomes [6]. For online learning, some of these challenges are related to students’ fatigue from synchronous teaching [7, 8], students’ negative perception of online courses [9], and students’ difficulties in emulating in-person experiential learning [10, 11]. For both in-person and online learning, some challenges are related to students’ negative responses to pedagogical innovations [2, 12–14] and students’ lack of understanding of the purpose of a new teaching and learning methodology and assessment [15].

The number of challenges instructors face has increased with technological advances and online education growth [15, 16]. To overcome these challenges and adequately prepare students to meet the demands of the work environment, which requires a breadth of skills from graduates, some adaptations in teaching and assessment approaches are required. Regarding assessments of students’ learning, instructors can consider assessments focusing on experiential learning (e.g., discussion of case studies, laboratory practices) [17, 18] and assessments enhancing participation in the classroom [19]. These assessments can supplement instructors’ existing approaches, often traditional methods focusing on conceptual learning (e.g., written exams and quizzes) [20], which have remained dominant even with the surge of assessment strategies enhancing experiential learning, which involves diverse hands-on activities such as laboratory experiments, fieldwork assignments, and field trips to promote critical thinking [9].

Understanding students’ preferences for different assessments is critical when choosing the proper pedagogical approaches that support students learning and overcome current challenges in higher education. Moreover, by incorporating students’ opinions in adopting pedagogical strategies, especially regarding assessment formats, instructors can enhance the confidence, engagement, and learning potential of students [21–23] and increase academic success [22–25].

Yet, our understanding of students’ opinions on learning assessments for both in-person and online learning environments is limited. Furthermore, evidence about students’ preferences for multiple assessment alternatives enhancing experiential learning is scarce. The objective of this study, therefore, is to determine students’ preferences for multiple assessment alternatives and attributes employed by educational programs that rely on experiential learning methodologies. This study answers the following questions: (1) Which assessment formats are preferable to students? and (2) If there is heterogeneity in preferences for assessment formats, can it be explained by observable student characteristics?

This research offers three contributions to the existing literature on the scholarship of teaching and learning. First, while students’ choices of schools, majors, and teaching methodologies have been examined, students’ preferences for assessment formats are overlooked. There are a few exceptions. Previous work has evaluated students’ preferences in specific fields, such as education and business degrees [26, 27] or preferences related to a few particular methods [28, 29]. To the best of our knowledge, students’ preferences for a wide range of assessment formats, particularly preferences for experiential learning assessments employed in an online classroom, have not been evaluated. As online instruction requires different strategies, it is unclear whether preferences for in-person assessments are similar to preferences for online evaluations.

Second, past studies have evaluated preferences for specific formats using traditional approaches such as approve/disapprove questions or rating scales such as Likert scales [23, 30]. Because it is difficult to ascertain the relative preferability of multiple approaches, traditional approaches might not be adequate to study preferences for various assessments, even among those with the same learning objective. This study employs the best-worst scaling (BWS) approach. BWS technique offers three advantages over traditional methods. Unlike conventional approaches where respondents can rank all methods from “most important” to “least important”, BWS requires respondents to make trade-offs among alternatives [31]. Second, unlike rating scales, the process behind BWS prevents a bias effect on scale scores [32]. Finally, rankings from best-worst (BW) choices have a lower cognitive difficulty for participants relative to discrete choice experiments [33, 34]. BWS applications in the evaluation of students’ preferences are limited [35, 36]. None of these studies have focused on student preferences for multiple assessment formats employed in in-person and online instruction.

The third contribution of this study is the elicitation of students’ preferences for assessment formats (e.g., quizzes) and assessment attributes (e.g., frequency) by employing two similar BWS surveys. In addition to better elucidating the drivers of students’ preferences for specific assessment formats, this also allows analysis of the consistency between students’ formats of choice and their preferred attributes of assessments.

## Materials and methods

Two complementary surveys were developed and administered to explore students’ preferences for assessments: one explores preferences for assessment formats while the other explores preferences for assessment attributes (S1 File has the original survey questions in Spanish). Participants were randomly assigned to one of these two versions. Measuring students’ preferences for both assessment formats—that were previously exposed to students—and assessment attributes facilitates understanding of students’ preferences for different evaluation approaches as well as a test of preference consistency.

### Studied assessment formats

We study 13 different assessment formats, which can be grouped into three categories. The first assessments evaluate learning beyond a conceptual understanding (hereinafter experiential learning-based assessments). They differ in terms of their outcome, sociability, and preparation time. For instance, case studies do not always require delivering a product as they are usually formative assessments that promote discussion [17]. In contrast, projects and portfolio development require the provision of a product at the end of the course [18]. Regarding sociability, delivering a final project often involves team collaboration, whereas portfolio development and lab sessions are conducted individually [37]. These last two require short investments of time per outcome; however, the total amount of time invested by the student can be significant if they are continuous throughout the semester.

Other assessments support class participation and students’ involvement in group activities, often through (intra-group) peer evaluation. Evaluating class participation encourages students’ attendance and participation in classroom discussions. In contrast, peer evaluation based on a teacher’s benchmark can incentivize group collaboration and mitigate free-rider problems in group-based assignments [19]. However, these assessments can be seen as a paternalistic approach to induce students’ involvement whose learning goals might not be apparent to students [21, 38].

Last, traditional assessments such as written exams and quizzes often test conceptual understanding and are simple to develop and implement. Compared to experiential learning-based assessments, less ambiguity and subjectivity are involved during evaluation and grading [39]. Some differences among them are related to frequency and content volume. While exams are summative assessments, quizzes are formative assessments based on open-ended questions or multiple-choice questions. Compared to quizzes of open-ended questions, multiple-choice tests can deliver objective feedback and mitigate trivial computational errors [20]. However, they can provide unintended corrective feedback and promote random guessing [20]. Regarding traditional summative assessments, open-book exams evaluate knowledge application, while proctored exams evaluate conceptual learning by supervising students’ activities during evaluation to prevent cheating [40, 41]. However, because proctored exams employ special software to monitor students during online evaluations (e.g., video recording), privacy comes into concern [42].

### Survey design

Tables 1 and 2 list the assessment formats and attributes identified for this study. Each table presents a brief description of each of the alternatives provided to the student during the survey. Alternatives were selected to reflect the main assessment formats or attributes widely implemented in applied sciences, including arts and music, life and social sciences, and medicine [3, 27, 43]. Table 1 also reflect assessment formats with which respondents are familiar from the beginning of their education. Alternatives presented in the surveys were validated by a small group of students (n = 10) who also pretested the survey.

**Table 1:** Student assessment formats (English translation).

Assessment Format	Description
Final Project	Involves considerable analysis and dedication from students.
	Instructions are generally delivered at the beginning of the semester. It has an important weight in the final course grade.
	Assigned to each student or group.
Class participation	Participation of students in the development of the classes is evaluated.
	Evaluation is continuous during the semester.
	Each student is evaluated.
Homework assignments	Take-home assignments are evaluated.
	Delivery times are usually short, and evaluation is continuous during the semester.
	Generally assigned to each student.
Analysis and discussion of case studies	Seeks to reflect what has been learned based on case study analysis and is materialized in a written document or oral presentation.
	Evaluates knowledge of the subject, good writing, and coherence.
	Assigned to each student or group.
Written essay	Tests knowledge about a specific topic and the student’s opinion is evaluated.
	Considers the use of information (theory) to make judgments.
	Assigned to each student.
Portfolio	Consists of a compilation of work that the student delivers on a recurring basis and includes works such as written documents, manual work, and audiovisual records.
	Evaluation can be continuous during the semester or at the end of the semester.
	Assigned to each student.
Continuous quizzes of multiple choice	Consists of a questionnaire containing a limited number of answer options.
	Each question assesses a single skill and/or content.
	Assigned to each student.
Continuous quizzes of open-ended questions	Each question assesses more than one skill or content and can be done in written or oral mode.
	Measure knowledge based on the student’s own responses.
	Assigned to each student.
Open book exam	Contains analysis questions that cannot be easily found in any source (internet and books).
	Measures deep knowledge through the application of what has been learned.
Professional presentations	Related to a specific topic using audiovisual materials.
	Assess knowledge of a topic and communication skills.
	Assigned to each student or group.
Proctored exam	It is time limited.
	Involves monitoring and supervising students during the exam.
	Assigned to each student.
Peer evaluation in group activities	Involves feedback given by peers based on a series of criteria, such as collaboration and effort in activities conducted within a group.
	Evaluation can be continuous during the semester or at the end of the semester.
Lab practices and simulations	They are based on practical activities.
	Allow students to rehearse technical skills.
	Assigned to each student or group.

**Table 2:** Student assessment attributes (English translation).

Assessment attribute	Description
Fast	Involves little time in the realization and preparation.
Valid	Appropriate for assessing the achievement of learning objectives.
Safe	Considers preventive measures to free the evaluation from cheating, fraud, and guessing the answers.
Precise	Easy to understand, with little ambiguity.
Pertinent	Reflects the actual level of knowledge of the student.
Simple	Easy to perform, and the task/activity/question is familiar to the student.
Realistic	Develops professional skills transferable to the real world.
Analytical	Promotes analysis, discussion, and debate.
Promoter	Promotes active student participation.
Driving	Develops different types of skills such as creative thinking, critical thinking, problem-solving, etc.
Strategic	High probability of getting good grades.
Frequent	Recurring during the semester.
Collective	Involves group activities and evaluations.

For each survey version, to determine the relative importance that students place on presented alternatives (formats or attributes depending on the survey version) based on the contribution towards students’ learning, we constructed a Case 1 BWS experiment [44, 45]. A Balanced Incomplete Block Design (BIBD) determined the allocation of the 13 alternatives to each question [46]. The design resulted in 13 choice questions, each including four alternatives (or options). The BIBD is one of the most used designs used in the BWS literature due to its desirable properties: it is balanced and orthogonal [47, 48]. In each survey application presented in this study, each alternative appeared four times across the BW choice questions and each pair of alternatives appeared once.

The order of the BW questions was randomized to avoid ordering effect bias. For each question in the BWS, respondents were asked to select, based on the alternative’s contribution to their learning, one alternative as best (most important) and one alternative as worst (least important) over all other options. Preceding each question were the following instructions (English translation): *“Next*, *we will present you 13 scenarios of 4 alternatives each*. *In each scenario*, *you must choose the option that you consider “most important” and the option you consider “least important” in facilitating YOUR LEARNING*. *In answering each question*: *(i) remember that the alternatives reflect those employed in your courses of applied sciences in your university*, *(ii) imagine that you can choose the alternative that facilitates YOUR LEARNING*, *and (iii) choose based on what you think*, *not what you think is most common or feasible in your career”*.

**Fig 1:**
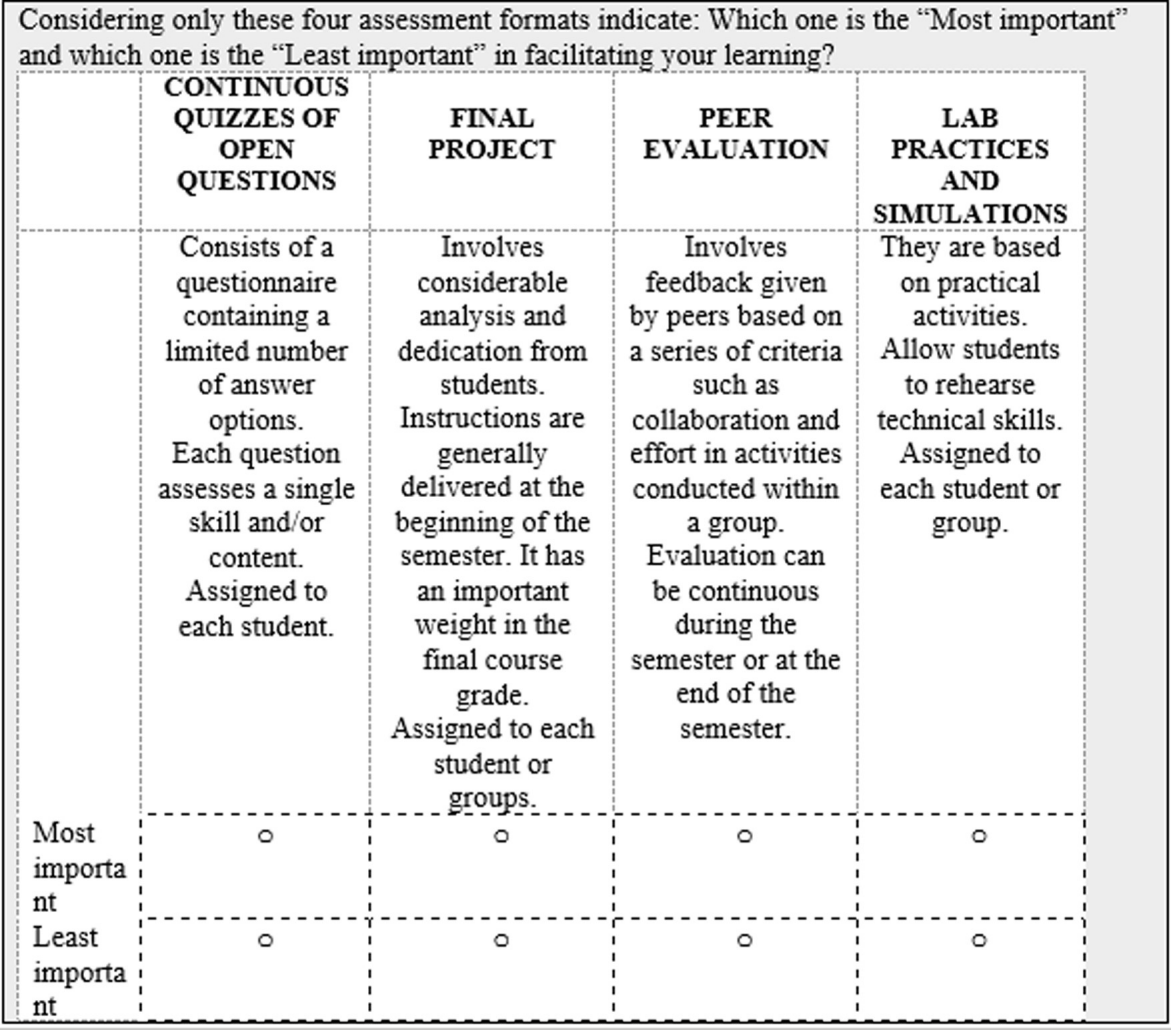
Example of a BW question in the survey version assessing preferences for assessment formats (English translation).

**Fig 2:**
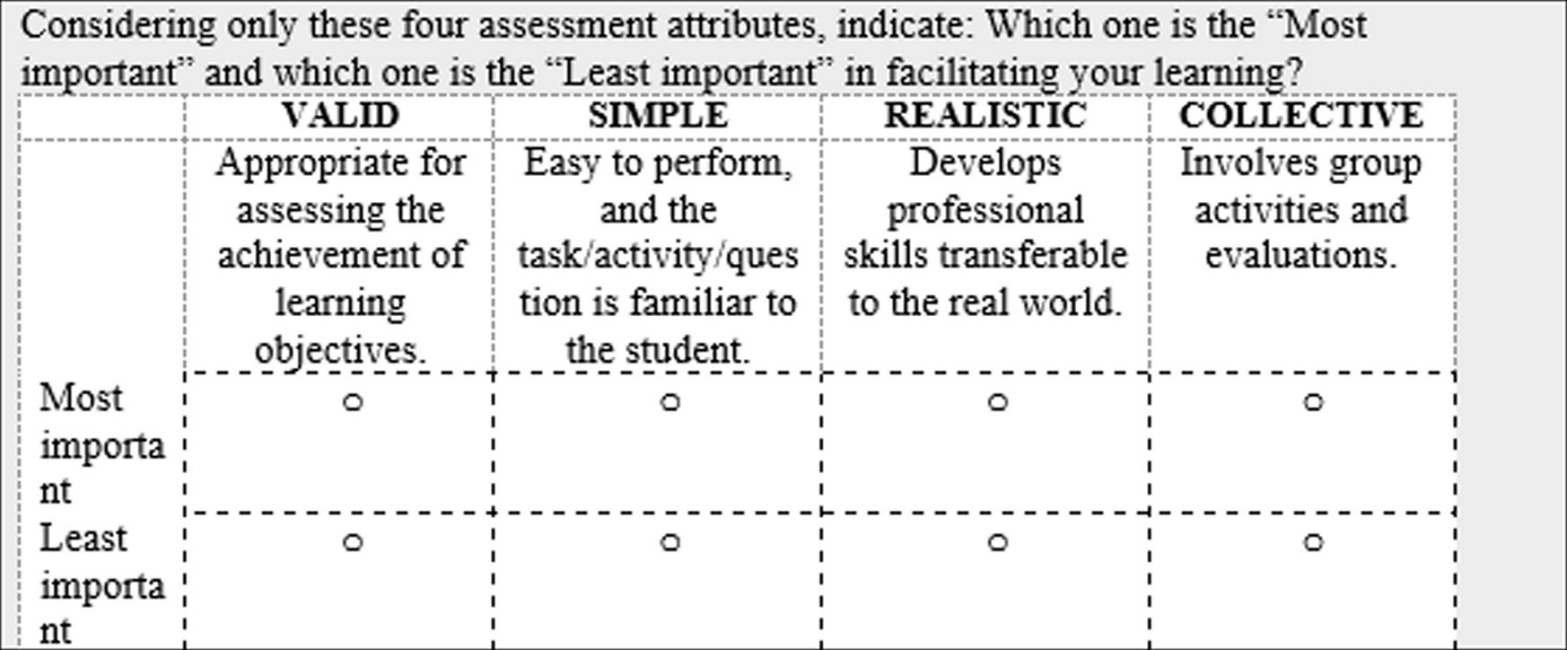
Example of a BW question in the survey version assessing preferences for assessment attributes (English translation).

In the BW questions and throughout the survey, we replaced the term *alternatives* with formats or attributes depending on the survey version respondents received. Examples of one of the questions in the BWS used in each survey version are presented in Figs 1 and 2. Before the BW questions, participants were provided with one example of a BW question. As a quality check, the options “most important” and “least important” in each task were not mutually exclusive; thus, participants were able to select any alternative as both “Most important” and “least important”. The one respondent who made this election was removed from the analysis.

The BWS approach has several advantages over other ranking and rating methods, such as providing better discrimination and being free from scale use bias [32]. However, it has the limitation of being less straightforward and/or more time-consuming than traditional ranking methods.

Hence, in both survey versions, respondents were asked the following question: *Which assessment alternatives do you consider to be “very important” to facilitate your learning*? For this question, a list of all options displayed in Table 1 for assessment formats (or Table 2 for assessment attributes) was then shown, and respondents were asked to provide their opinion on each option. Responses to this question offer direct binary responses of preferences that can be compared with the BW responses to test consistency.

The second part of the two surveys contains questions that investigate how students’ perceptions of the various assessment formats (or attributes) relate to (i) expected grades, (ii) students’ self-reported learning style and personality type, and (iii) responses to a more straightforward question on which students indicate their most preferred alternatives to support their learning from all alternatives (formats or attributes). Answers to this last question provide a convergent validity test. Responses to these questions are the focus of another study.

The last part of the surveys contains exit questions that include conventional sociodemographic questions that previous work has identified to be correlated with preferences for teaching styles such as major, year of college, gender, etc. [49]. In the study, the respondent population was comprised of a homogeneous group of students. Thus, many of the typical demographic variables, such as age, were excluded from the analysis since they contribute little explanatory power. The very last question serves as a data integrity check as respondents were asked to indicate how many days are in a week (options ranging from 1 to 7). Everyone accurately answered the data integrity check question. As shown in the data analysis section, students were inconsistent in some questions (e.g., BW responses), which indicates that this type of question does not guarantee respondents were paying attention throughout the survey.

### Data

The data were collected through an online survey, which was completed by 218 students (106 completed the assessment formats survey and 112 completed the assessment attributes surveys) from the Pontificia Universidad Catolica de Chile in July 2021. In total, 450 students received an email with the link to the survey. Of the 112 (120) participants who started and completed the assessment formats survey (or assessment attributes), 106 (112) completed the BW questions as well as the personality and learning style questions; these participants composed the final sample in the surveys lasting approximately 25 minutes. Students have shifted from in-person to online classes at this university due to the COVID-19 outbreak since March 2020. Therefore, exploring these students’ opinions was important for this study as they reflect the views of those who participated in online evaluation for multiple semesters.

Only students enrolled in majors whose courses expose students to experiential learning activities were invited to participate in the study. Invitations were extended during virtual class sessions via zoom with the consent of the instructor; those participants who expressed interest in participating were asked to provide their email and/or fill out a Google survey that requested their email. A list of potential participants was created based on this procedure. This was the protocol approved for this study by the Ethics Committee of Pontificia Universidad Catolica de Chile (Protocol ID 210430001). Students from this list were then randomly assigned to one of the two survey versions. Email invitations with the corresponding survey link were sent to each student in the list with three reminders to complete the survey. Only those students who provides written consent (those who agreed to participate by clicking continue after presenting them information about their voluntary participation), could start the survey. As compensation, participants were told that they would have a chance to win a gift card worth approximately USD 20.

Table 3 summarizes the characteristics of the sampled students. According to the results from the survey, about 63% of participants were female students, and nearly 25% were first-generation college students. In terms of the number of years in school, 16% were first-year students, 24% second-year students, 20% third-year students, and the remaining 41% were in their fourth year or above. Most of the respondents were enrolled in Medicine, Agronomy, and Engineering, with shares of 26%, 21%, and 18%, respectively.

**Table 3:** Sample characteristics.

Variable	Definition	Total	Survey Sample	
		(%)	Attributes	Formats
			(%)	(%)
Female	1 if female, 0 otherwise	63	65	60
First generation	First generation studying in college	24	19	29
Year	First year	16	19	12
	Second year	24	27	21
	Third year	20	18	22
	Fourth year or above	41	37	45
Major	Agronomy	21	21	22
	Medicine	26	28	23
	Engineering	18	19	16
	Psychology	8	8	8
	Biology	2	2	3
	Chemistry	7	4	10
	Arts	3	1	6
	Education	5	5	5
	Other	10	12	8
Observations		218	112	106

Furthermore, we wanted to get a sense of the respondents’ personality and learning style as they may impact how students feel towards teaching methods [50, 51] and assessments [26, 28, 29]. Based on the description of five personality factors prevalent in educational psychology: extroversion, agreeableness, conscientiousness, neuroticism, and openness to new experiences [52], we asked respondents to choose the description they identify the most (or the least) with [50]. Specifically, students were provided a description of each of 5 personality factors and were asked to choose which one they feel describes them the most (the least). Generic labels were given to each option (e.g., personality type A) instead of actual names (e.g., responsibility). A similar procedure was used for the learning style questions. S1 Fig indicates that conscientiousness, characterized by responsibility, reliability, and organization, is the personality trait representing most of the respondents (32%), followed by agreeableness, which is associated with honesty and courtesy (31.6%).

Similarly, based on self-reported measures of four learning styles [53], we asked respondents to choose the description of the learning style they identified with the most (or the least). S2 Fig indicates that theoretical learners characterized for thinking sequentially and using logic for problem-solving had the highest representation of respondents (41%). The reflective (24%), on the other hand, based their learning on data gathering and analysis. The pragmatic students (16%) prefer dynamic discussions and easily posit ideas and put them into practice. Lastly, active learners (20%) prefer short-term plans and team-based activities and show enthusiasm for new activities. Interestingly, 14% of respondents indicated that openness to experience, characterized by imagination and preference for variety, was the dominant personality factor.

### Analytical model

Based on how the BW questions were framed to participants (Figs 1 and 2), we formatted or exploded the BW responses into choice sets based on the maxdiff (or paired) model [44]. This choice process model assumes that respondents evaluate all possible pairs of options and simultaneously choose the pair of options that maximizes the difference between the best and the worst choices [54].

To analyze the data exploded according to the maxdiff model, we employ discrete choice models. These analytical models are based on random utility theory [55]. That is, they assume that the indirect utility of a respondent derived from the selected alternative in a BW question is defined by a deterministic utility component plus a stochastic error term.

As heterogeneity in student preferences for assessments is expected, first, we employ a mixed logit model (MXL) for panel data [56]. Then, to identify possible sources of heterogeneity, we estimated a latent class conditional logistic (LCL) model. This model creates *C* segments or classes of students with similar taste parameters and characteristics to account for differences in preferences [57]. We jointly estimate class membership and choice preferences as a function of individual characteristics in a LCL model using the expectation maximization (EM) algorithm [58, 59]. Details for the econometric analyses are in the S2 File.

To measure the extent of the relative importance between alternatives, we calculated the share of preferences (SP) for each alternative using the estimated parameters from MXL and LCL models (predictions were conducted separately for each class) as follows: ${SP}_{i}=\frac{\exp\left({\hat{\beta }}_{nit}\right)}{\sum_{p=1}^{J}\exp\left({\hat{\beta }}_{npt}\right)}$. These shares across the 13 alternatives sums to one. The parameters of the MXL and LCL models were estimated using STATA (17.0) and standard errors of the SP were computed using the delta method.

## Results and discussion

### Which assessments are valuable to students?

To further explore heterogeneity in student preferences, Tables 4 and 5 report the estimates from the MXL model for assessment formats and attributes. The coefficients in column 1 (in Table 4) reflect the relative importance of each of the 12 assessments relative to the proctored exam (attribute strategic), which was normalized to zero for identification purposes as it was the least important assessment, based on the B-W scores in S1 Table (S2 Table). As expected, most coefficients have a positive and statistically significant sign. In Table 4, the coefficient of peer evaluation is not statistically significant, which indicates that it is not preferred over proctored exam. The standard deviation coefficients of each alternative in Tables 4 and 5 are highly significant, confirming that heterogeneity characterizes students’ preferences for assessments.

**Table 4:** Maximum likelihood estimates from mixed logit model and share of preferences of assessment formats.

Assessment Formats	Estimates		SP	Rank
	Mean	SD		
Final Project	2.074***	1.457***	7.482***	4
	(0.172)	(0.175)	(1.138)	
Class participation	0.484**	1.587***	1.526***	11
	(0.178)	(0.203)	(0.266)	
Homework assignments	0.647***	1.508***	1.795***	10
	(0.174)	(0.160)	(0.307)	
Analysis and discussion of case studies	3.389***	1.252***	27.863***	2
	(0.198)	(0.165)	(3.427)	
Written essay	0.959***	0.903***	2.454***	8
	(0.147)	(0.131)	(0.336)	
Portfolio	1.193***	2.069***	3.099***	7
	(0.185)	(0.200)	(0.541)	
Continuous quizzes of multiple choice	0.676***	1.520***	1.849***	9
	(0.190)	(0.228)	(0.316)	
Continuous quizzes of open questions	1.921***	-0.626***	6.421***	5
	(0.150)	(0.169)	(0.815)	
Open book exam	1.534***	0.723***	4.359***	6
	(0.145)	(0.148)	(0.567)	
Professional presentations	2.114***	1.731***	7.787***	3
	(0.187)	(0.174)	(1.309)	
Peer evaluation	-0.117	1.928***	0.837***	13
	(0.191)	(0.173)	(0.160)	
Lab practices and simulations	3.576***	1.968***	33.588***	1
	(0.206)	(0.237)	(3.905)	
Proctored exam			0.940***	12
			(0.127)	
Log likelihood	-3003.283			
N	12613			
Likelihood Ratio Test Chi-squared (12)	1099.675			

***Notes*:** Standard errors in parentheses. Levels of statistical significance

’ 0.1

* 0.05

** 0.01

*** 0.001.

**Table 5:** Maximum likelihood estimates from mixed logit model and share of preferences of assessment attributes.

Assessment Attributes	Estimates		SP		Rank
	Mean	SD			
Valid	2.116***	-0.683***	2.787***		7
	(0.160)	(0.163)	(0.426)		
Safe	0.488*	1.601***	0.547***		11
	(0.222)	(0.197)	(0.125)		
Precise	2.334***	0.960***	3.467***		6
	(0.169)	(0.178)	(0.524)		
Pertinent	3.263***	1.189***	8.774***		3
	(0.187)	(0.163)	(1.353)		
Simple	1.147***	1.303***	1.058***		9
	(0.176)	(0.215)	(0.188)		
Realistic	4.399***	1.198***	27.334***		2
	(0.206)	(0.230)	(3.443)		
Analytical	3.190***	-1.180***	8.157***		4
	(0.183)	(0.198)	(1.229)		
Promoter	2.452***	1.148***	3.899***		5
	(0.179)	(0.187)	(0.641)		
Driving	4.808***	1.446***	41.125***		1
	(0.231)	(0.196)	(4.616)		
Strategic	0.450*	1.654***	0.527***		12
	(0.200)	(0.218)	(0.107)		
Frequent	0.913***	1.530***	0.837***		10
	(0.189)	(0.178)	(0.161)		
Collective	1.233***	1.623***	1.153***		8
	(0.186)	(0.191)	(0.224)		
Fast			0.336***		13
			(0.054)		
Log-likelihood	-2426.378				
N	17472				
Likelihood Ratio Test Chi-squared (12)	527.163				

***Notes*:** Standard errors in parentheses. Levels of statistical significance

’ 0.1

* 0.05

** 0.01

*** 0.001.

**Fig 3:**
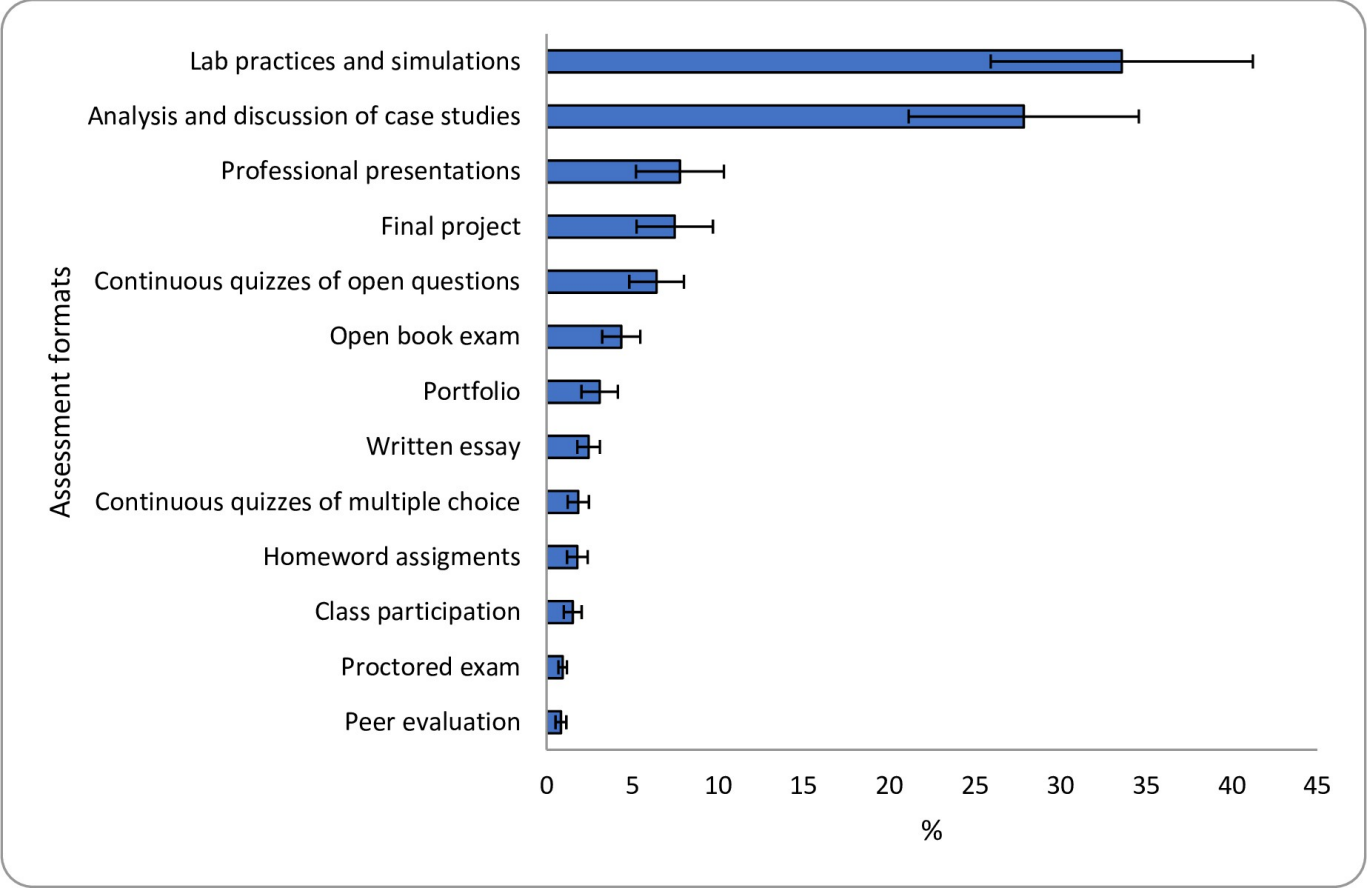
Share of preferences (SP) for assessment formats. SP for each alternative was calculated based on the MXL parameters. The error bars represent 95% Confidence Intervals (CI).

**Fig 4:**
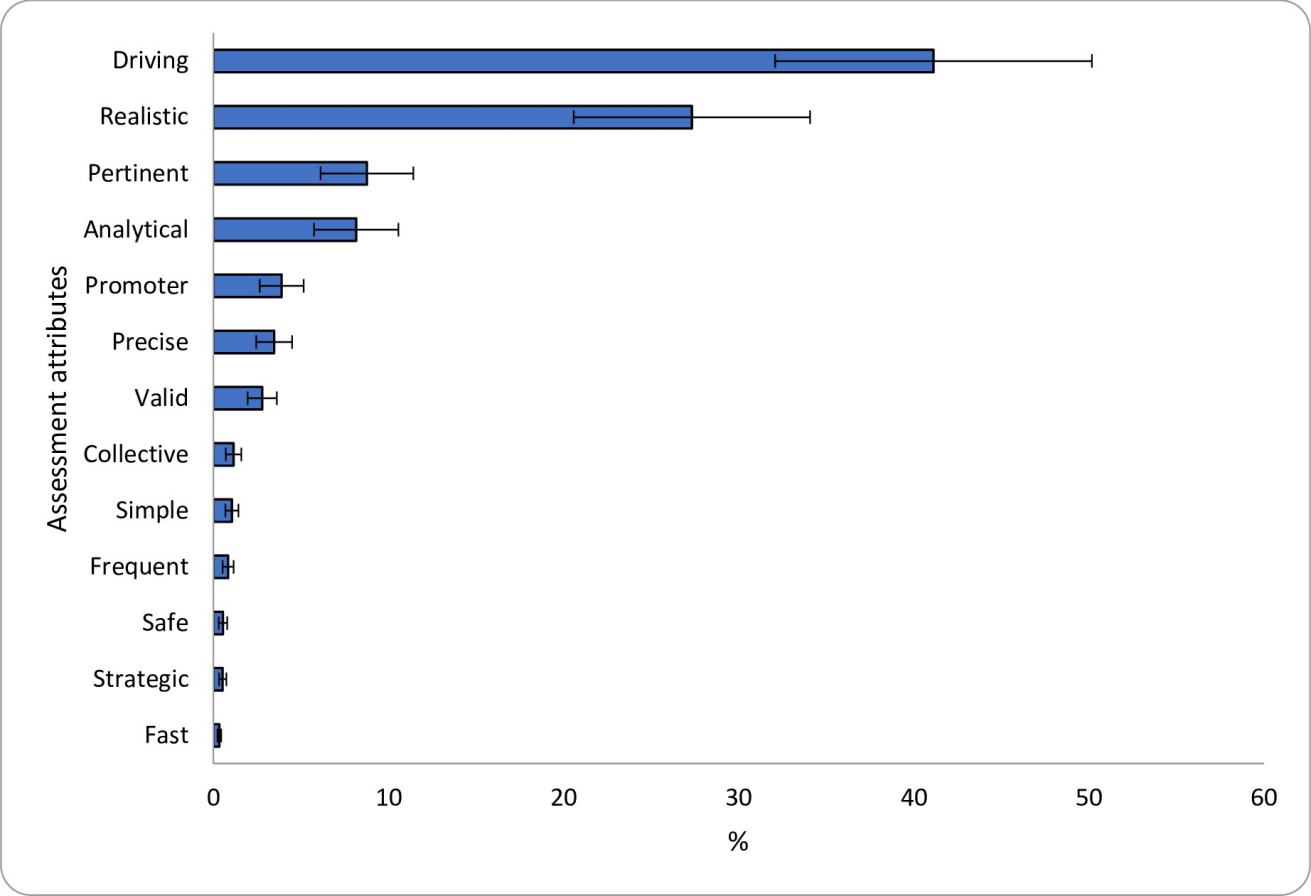
Share of preferences (SP) for assessment attributes. SP for each alternative was calculated based on the MXL parameters. The error bars represent 95% Confidence Intervals (CI).

To provide a more intuitive interpretation of the results, Fig 3 (Fig 4) reports the share of preferences (SP) for the different assessment formats (attributes). Results reveal that participating in lab practices and simulations (presented to participants as practical activities that support technical skills rehearsal) and analyzing and discussing case studies (subjects were told that knowledge of the subject, good writing, and coherence are evaluated) were the most and second most desirable options that contribute to student learning as 34% and 28% of respondents view them as the most important format respectively. Given that participants are from careers that require students’ real-world skills, it might not be surprising that learning through solving case studies or through lab practices and simulations is valuable to them.

Professional presentations are considered the third most important assessment by 8% of the respondents, as shown in Table 1, professional presentations entail using audiovisual materials to assess knowledge of a topic. There is a strong emphasis that in addition to understanding an academic subject, competencies such as communication and presentation skills are important for graduates to have. Yet, professional presentations were nearly four times less important for students than assessments that evaluate other skills such as analytical ones (e.g., analysis and discussion of case studies analysis that evaluate critical thinking, 8 vs. 28%).

A final project that involves considerable analysis and dedication from students, particularly at the end of the semester, is considered the fourth-most important assessment by 7% of the respondents. Compared to other summative evaluations (e.g., exams), a final project is a more straightforward measure to evaluate mastery of a subject [43]. However, completing a final project might demand more time and effort from students; therefore, fewer students prefer them compared to other performance assessments such as lab practices (7% vs. 34%).

Moreover, continuous quizzes of open-ended questions are considered the fifth most important assessment, with 6% of the participants choosing this option. Compared to quizzes of open-ended questions, quizzes of multiple-choice questions, ranked in ninth place, were considered three times less important (6.4% vs. 1.9%). This indicates that students prefer tests that give them the flexibility to answer and demonstrate their knowledge on the subject, even at the expense of receiving less objective feedback [20]. This finding contrasts earlier results indicating that students favor multiple-choice format exams to open-ended questions [27].

Compared to open-book exam, the sixth most important assessment, proctored exam is considered four times less important (4% vs. 1%). This is in line with a previous study reporting a dislike among students for supervised and intrusive evaluations [60]. Even fewer respondents (3%) see a portfolio, which indicates that among all less conventional assessments, portfolio is the least preferred.

Between 1.8% and 2.5% of respondents see homework assignments, multiple-choice tests, and written essays as valuable assessments for learning outcomes. This highlights students’ low inclinations towards traditional evaluations, especially written ones. Compared with more innovative assessment methods (e.g., presentation, case-based evaluations), traditional evaluation modes (e.g., quizzes) can be perceived as less accurate and fair measures of learning [27].

Interestingly, class participation is only preferred by 1.5%. Evaluating class participation can be perceived as a subjective and paternalistic measure, placing class participation as one of the least preferred alternatives. Similarly, peer evaluation that involves engagement from students in their groups is not statistically different from proctored exam, and it is considered along with proctored exam the least preferred assessment, only important to 0.8% of the respondents. Instruction aspects such as clarity of the goals, the extent of independent learning, and workload appropriateness are critical in supporting learning [61]. It is possible students perceive class participation and peer evaluation as lacking in one or more of these aspects, which might explain low students’ inclinations towards the evaluation of class participation and peer evaluation. Overall, the results indicate that, in line with the description of the alternatives given to the participants, respondents are less inclined towards time-limited and supervised evaluations.

Most notably, the preference ranking of assessment formats correlates with that assessment attributes, as shown in Fig 4. For example, in line with the description of attributes provided to participants (description in parentheses), more than two-thirds of respondents would prefer assessments with attributes such as driving (develops different types of skills such as creative thinking, critical thinking, problem-solving, etc.) and realistic (develops professional skills transferable to the real world). These findings are consistent with the ranking of students’ preference shares of assessment formats (Fig 3) that placed lab practices and simulations and case studies as the most important assessments.

In addition, Fig 4 shows that fast (involves little time in the realization and preparation.), strategic (high probability of getting good grades), safe (considers preventive measures to free the evaluation from cheating, fraud, and guessing the answers), and frequent (recurrent during the semester.) commanded the lowest preference share among respondents with about 2% choosing these alternatives as the most important attributes. These results are in line with results in Table 4, showing that proctored exam and continuous quizzes of multiple-choice questions had one of the lowest share of preferences among respondents (2.8%). Other attributes such as simple (easy to perform, and the task/activity/question is familiar to the student) and collective (involves group activities and evaluations) also commanded a low preference share among respondents, with about 2% choosing these alternatives as the most important. This is also consistent with the results in Fig 3, showing that peer evaluation and class participation were considered among the assessment formats with the lowest preference share (2.4%).

The results in Fig 4 also show, in combination with the results in Fig 3, that priority is given to assessments that are pertinent (reflects the actual level of knowledge of the student) and analytical (promotes analysis, discussion, and debate) by nearly 17% of respondents in the survey of assessment attributes (for instance, professional presentations and final project, are considered as the most important assessment formats by nearly 16% of respondents).

As a whole, the results show concordance between preferences elicited in both surveys: the survey eliciting preferences for assessment formats and the survey eliciting preferences for attributes of assessments. Moreover, it provides a robustness check for our elicitation approach.

### Can heterogeneity in preferences be explained by observable student characteristics?

We used the LCL model to understand the heterogeneity in preferences indicated by the SD of the MXL estimates (Tables 4 and 5). For the BW responses related to assessment formats (assessment attributes). We analyzed models with up to eight latent classes and selected the optimal class number given the BIC and CAIC values (S3 Table).

From the results of the maximum likelihood (ML) estimations for assessment formats (S4 Table) and attributes (S5 Table), we can infer that there are important differences in the preference ranking of alternatives across classes. That is, although lab practices and simulations have the highest preference share in all classes of respondents, except for class 1 respondents (who prefer case studies over lab practices), the relative importance of the most preferred alternative compared to other options varies across classes (S4 Table). Similarly, driving is the preferred option for all respondents, except for class 1 respondents who consider realistic and pertinent more critical attributes than driving (S5 Table).

LCL model results further show that respondents’ demographic variables have a minor role in explaining class membership. This highlights the complexity of having *a priori* segments of students to target in the evaluation of learning. These results are in line with previous findings indicating no influence of gender and age on students’ opinions about online instruction and assessments [30, 62].

### Robustness

We conducted three additional analyses to check the consistency of our results. First, we compared the BW responses with a direct question eliciting students’ preferences regarding assessment formats and attributes. Students’ preferences—measured by individual-specific BW responses (S1 and S2 Tables) and estimated preferences (Figs 3 and 4)—agree with responses to the direct question (S3 and S4 Figs). This is the case, for the most important and least important alternatives. For those alternatives in the middle in the preference ranking, responses are different. This is expected as the BWS requires respondents to make trade-offs [31].

Second, we examined Pearson correlations between individual-level preferences. Consistent with regression results, we found negative associations, which were moderate [63], between assessments, ranked higher in the preference rank (e.g., presentations) and more traditional assessments ranked lower in the preference rank, such as exams (S6 Table). For instance, presentations were negatively correlated with continuous quizzes with open questions (*ρ* = −0.39), open book exam (*ρ* = −0.36), and proctored exam (*ρ* = −0.38). There were also negative (moderate) correlations between preferences for assessment attributes between attributes found more desirable, such as driving and pertinent and attributes found less preferable, such as simple and collective (S7 Table). For instance, there were negative associations between simple and driving (*ρ* = −0.37), and between pertinent and collective (*ρ* = −0.40).

Last, respondents’ learning styles and personality traits were excluded in regression analysis because (i) they were correlated among each other and with students’ characteristics (e.g., gender), which made it challenging to study the role of both learning style and personality in influencing preferences using the LCL model. To overcome these challenges and further investigate whether they explain preferences for assessments and attributes, we examine Kendall’s tau correlations [45] between students’ individual-level preferences (S1 and S2 Tables) and their self-reported learning styles and personality traits. Overall, preferences for assessments and attributes were weakly correlated with personality (S8 and S9 Tables, respectively) and learning style (S10 and S11 Tables, respectively). Moreover, the results indicate few Kendall’s tau correlation values that were statistically significant (*p<*0.05). Because values between 0.10 and 0.19 indicate weak associations [64], our results provide only limited support for correlations. For instance, conscientiousness was only weakly correlated with preferences for homework assignments (*τ* = 0.12) (S8 Table).

## Conclusions and implications

Our results indicate that students have a clear preference for supporting experiential learning-based assessments, such as lab activities and simulations or analysis of case studies, and a strong dislike for assessments that involve supervised and time-constrained assessments (e.g., proctored exams). This finding is especially important for educators as it indicates that traditional assessments (e.g., homework assignments) are less likely to find support among students compared to assessments that evaluate experiential learning (e.g., case studies).

Instructors would like to have observable a *priori* segments of students to target. Our results show that because of the complexity of students’ preferences, such a priori segmentation might not be possible. The results from the latent class analyses show little of the segment differences can be predicted using observable demographics such as gender or academic year (S4 and S5 Tables). Whether this is possible with an expanded set of demographic and behavioral variables is an empirical question.

One caveat of our study is that in the survey, we did not ask respondents about their prior experience with assessment alternatives. Therefore, we are unable to determine how students’ previous exposure to the studied assessment methods affects our results. Because we presented a description of each assessment evaluated in this study and we pre-tested assessment alternatives with students, we expect that students understood the options presented to them during the survey.

How can instructors use our results without a complete overhaul of a curriculum? Our findings indicate that students also value professional presentations and final projects, which can be simpler to apply and prepare for both students and instructors compared to the more popular assessments (e.g., case studies and lab practices). Considering that there were some students who prefer other assessments (e.g., open book exams), instructors could adopt different options instead of choosing only laboratory experiments or case studies. Furthermore, as traditional assessments have unique advantages over experiential learning-based assessments (e.g., less ambiguity is involved when grading, implementation and preparation are convenient, etc.), instructors might be better off employing one or more traditional assessments valued by students in applied sciences rather than eliminating them from the curriculum.

How can instructors conveniently adapt their evaluation approach? There are some key aspects instructors might consider if they decide to adapt their assessments. First, experiential learning-based assessments can involve nontrivial preparation time. However, once they are developed, the preparation time will be shortened. They often involve unique solutions; therefore, instructors do not have to update questions each semester as they do with traditional evaluations (to prevent students from using solutions from past evaluations). Second, non-traditional assessments can involve considerable grading time. Instead, instructors can implement formative assessments without grading. In this case, instructors can employ a rubric and use students’ peers to provide prompt and adequate feedback [65, 66]. In other circumstances, a well-designed rubric can be used by teaching assistants for grading [67].

The present study lays the groundwork for future research into student evaluation methods employed in both in-person and online instruction in higher education. Further research could focus on how students’ preferences change in light of a different set of alternatives or over time. There were attributes for assessments such as promoter, precise, and valid that were valuable only to some students. It is possible that in the absence of alternatives such as driving and realistic, ranked high by most students, preferences for such attributes would have been higher. Thus, further investigation is necessary to better understand preferences for alternatives in the middle of the preference ranking. As instructors and students get familiar with new teaching and assessment methods, students’ preferences might evolve. Therefore, another potential extension of this study is to collect multiple rounds of observations to see whether there are shifts in preferences, especially after students return to their in-person classes. Our findings may be used in future studies to understand the role of different assessments on students’ academic success.

## Supporting information

S1 Fig Self-reported personality trait of respondents. (DOCX)

S2 Fig Self-reported learning style of respondents. (DOCX)

S3 Fig Responses to the direct question on which students indicate their most preferred assessment formats from all alternatives. (DOCX)

S4 Fig Responses to the direct question on which students indicate their most preferred assessment attributes from all alternatives. (DOCX)

S1 Table Assessment format scores and their relative importance. (DOCX)

S2 Table Assessment attribute scores and their relative importance. (DOCX)

S3 Table Criteria for determining the optimal number of latent classes. (DOCX)

S4 Table Latent class model estimates and shares of preferences (SP) of assessment formats. (DOCX)

S5 Table Latent class model estimates and shares of preferences (SP) of assessment attributes. (DOCX)

S6 Table Pearson correlations between individual-specific B-W scores for assessment formats. (DOCX)

S7 Table Pearson correlations between individual-specific B-W scores for assessment attributes. (DOCX)

S8 Table Kendall’s tau correlations of personality traits with preferences for assessment formats. (DOCX)

S9 Table Kendall’s tau correlations of personality traits with preferences for assessment attributes. (DOCX)

S10 Table Kendall’s tau correlations of learning style with preferences for assessment formats. (DOCX)

S11 Table Kendall’s tau correlations of learning style with preferences for assessment attributes. (DOCX)

S1 File Questionnaires in Spanish. (PDF)

S2 File Econometric analysis. (DOCX)
