# Supplementary material for: How to assess? Student preferences for methods to assess experiential learning: A best-worst scaling approach
Source: PLoS One. 2022 Oct 27;17(10):e0276745. doi: 10.1371/journal.pone.0276745 (PMC9612489; doi:10.1371/journal.pone.0276745)
Supplement: S1 Fig — (DOCX) [file pone.0276745.s001.docx]

**S1 Fig.** **Self-reported personality trait of respondents**

***Note:*** Descriptions provided to participants were: *Type A* *[Conscientiousness]:* It is a multidimensional personality trait that integrates achievement orientation, dependability, and orderliness. Conscientious persons are characterized by organization and efficiency. *Type B [Agreeableness]:* Individuals who are agreeable, cooperative, caring, gentle, trusting, forgiving, compliant, and empathetic. *Type C [Openness to Experience]:* Individuals who are open to experience are untraditional, imaginative, and intellectually curious. *Type D [Extroversion]:* Individuals who are extroverts, enthusiastic, cheerful, assertive, energetic, sociable, and seek opportunities for excitement. *Type E [Neuroticism]:* Emotionally stable individuals stand out as being calm, self-confident, stress-tolerant, relaxed and even-tempered.

Names in brackets were not presented to participants.
