# Supplementary material for: How to assess? Student preferences for methods to assess experiential learning: A best-worst scaling approach
Source: PLoS One. 2022 Oct 27;17(10):e0276745. doi: 10.1371/journal.pone.0276745 (PMC9612489; doi:10.1371/journal.pone.0276745)
Supplement: S2 Fig — (DOCX) [file pone.0276745.s002.docx]

**S2 Fig.** **Self-reported learning style of respondents**

***Note:*** Descriptions provided to participants were:  *Type A [Active].* Individuals who involve themselves in new experiences, tackle problems by brainstorming and move from one task to the next as the excitement fades. *Type B [Reflective].* Individuals who tend to be cautious and thoughtful people who like to consider all the possible angles before making decisions and whose actions are based on observation and reflection. *Type C [Theoretical].* Individuals who integrate their observations into logical models based on analysis and objectivity. *Type D [Pragmatic].* Individuals who are practical people who get impatient with reflection, preferring to apply new ideas immediately.

Names in brackets were not presented to participants.
