# Supplementary material for: How to assess? Student preferences for methods to assess experiential learning: A best-worst scaling approach
Source: PLoS One. 2022 Oct 27;17(10):e0276745. doi: 10.1371/journal.pone.0276745 (PMC9612489; doi:10.1371/journal.pone.0276745)
Supplement: S4 Fig — (DOCX) [file pone.0276745.s004.docx]

**S4 Fig. Responses to the direct question on which students indicate their most preferred assessment attributes from all alternatives.** In this question, students can choose more than one alternative.
