# Supplementary material for: How to assess? Student preferences for methods to assess experiential learning: A best-worst scaling approach
Source: PLoS One. 2022 Oct 27;17(10):e0276745. doi: 10.1371/journal.pone.0276745 (PMC9612489; doi:10.1371/journal.pone.0276745)
Supplement: S1 Table — (DOCX) [file pone.0276745.s005.docx]

**S1 Table. Assessment format scores and their relative importance.**

| Format | B | W | B-W | sqrt (B/W) | Relative Importance | | Rank | B-W* | SD |
| --- | --- | --- | --- | --- | --- | --- | --- | --- | --- |
| Final Project | 132 | 59 | 73 | 1.5 | 8.4% | | 4 | 0.7 | 1.5 |
| Class participation | 48 | 142 | -94 | 0.6 | 3.3% | | 10 | -0.9 | 1.7 |
| Homework assignments | 49 | 177 | -128 | 0.5 | 3.0% | | 11 | -1.2 | 2.0 |
| Analysis and discussion of case studies | 239 | 14 | 225 | 4.1 | 23.3% | | 1 | 2.1 | 1.7 |
| Written essay | 50 | 109 | -59 | 0.7 | 3.8% | | 8 | -0.6 | 1.6 |
| Portfolio | 101 | 96 | 5 | 1.0 | 5.8% | | 7 | 0.1 | 1.5 |
| Continuous quizzes of multiple choice | 61 | 142 | -81 | 0.7 | 3.7% | | 9 | -0.8 | 2.1 |
| Continuous quizzes of open questions | 126 | 74 | 52 | 1.3 | 7.4% | | 5 | 0.5 | 1.6 |
| Open book exam | 82 | 69 | 13 | 1.0 | 6.1% | | 6 | 0.1 | 2.0 |
| Professional presentations | 163 | 60 | 103 | 1.7 | 9.3% | | 3 | 1.0 | 1.9 |
| Proctored exam | 29 | 213 | -184 | 0.4 | 2.1% | | 13 | -1.7 | 1.9 |
| Peer evaluation | 39 | 205 | -166 | 0.4 | 2.5% | | 12 | -1.8 | 2.1 |
| Lab practices and simulations | 259 | 18 | 241 | 3.8 | 21.4% | | 2 | 2.3 | 1.7 |
|  | | | | |  |  |  |  |  |

***Notes:*** * Denotes individual-level B-W scores. The relative importance of each alternative was calculated from the square-root scale values using the following formula: $\frac{sqrt\left( \frac{B}{W} \right)}{\sum_{j=1}^{J} sqrt\left( \frac{B}{W} \right)}*100\%$.
