# Supplementary material for: How to assess? Student preferences for methods to assess experiential learning: A best-worst scaling approach
Source: PLoS One. 2022 Oct 27;17(10):e0276745. doi: 10.1371/journal.pone.0276745 (PMC9612489; doi:10.1371/journal.pone.0276745)
Supplement: S2 Table — (DOCX) [file pone.0276745.s006.docx]

**S2 Table. Assessment attribute scores and their relative importance.**

| Attribute | B | W | B-W | sqrt (B/W) | Relative Importance | Rank | B-W* | SD |
| --- | --- | --- | --- | --- | --- | --- | --- | --- |
| Fast | 18 | 267 | -249 | 0.3 | 1.1% | 13 | -2.2 | 1 |
| Valid | 119 | 99 | 20 | 1.1 | 4.7% | 7 | 0.2 | 1 |
| Safe | 23 | 199 | -176 | 0.3 | 1.5% | 11 | -1.6 | 2 |
| Precise | 83 | 45 | 38 | 1.4 | 5.9% | 6 | 0.3 | 2 |
| Pertinent | 177 | 34 | 143 | 2.3 | 9.9% | 3 | 1.3 | 2 |
| Simple | 39 | 158 | -119 | 0.5 | 2.2% | 10 | -1.1 | 1 |
| Realistic | 272 | 5 | 267 | 7.4 | 31.9% | 1 | 2.4 | 1 |
| Analytical | 166 | 41 | 125 | 2.0 | 8.7% | 4 | 1.1 | 2 |
| Promoter | 132 | 54 | 78 | 1.6 | 6.8% | 5 | 0.7 | 2 |
| Driving | 311 | 13 | 298 | 4.9 | 21.2% | 2 | 2.7 | 2 |
| Strategic | 23 | 233 | -210 | 0.3 | 1.4% | 12 | -1.9 | 2 |
| Frequent | 52 | 184 | -132 | 0.5 | 2.3% | 9 | -1.2 | 2 |
| Collective | 41 | 124 | -83 | 0.6 | 2.5% | 8 | -0.7 | 1 |

***Notes:*** * Denotes individual-level B-W scores. The relative importance for each alternative was calculated from the square-root scale values using the following formula: $\frac{sqrt\left( \frac{B}{W} \right)}{\sum_{j=1}^{J} sqrt\left( \frac{B}{W} \right)}*100\%$
