# Supplementary material for: How to assess? Student preferences for methods to assess experiential learning: A best-worst scaling approach
Source: PLoS One. 2022 Oct 27;17(10):e0276745. doi: 10.1371/journal.pone.0276745 (PMC9612489; doi:10.1371/journal.pone.0276745)
Supplement: S3 Table — (DOCX) [file pone.0276745.s007.docx]

**S3 Table.** **Criteria for determining the optimal number of latent classes.**

| Formats | | | | Attributes | | | |
| --- | --- | --- | --- | --- | --- | --- | --- |
| LLF | Nparam | CAIC | BIC | LLF | Nparam | CAIC | BIC |
| -2754.38 | 28 | 5667.34 | 5639.34 | -2532.94 | 28 | 5226.00 | 5198.00 |
| -2700.56 | 44 | 5650.31 | 5606.31 | -2481.52 | 44 | 5214.66 | 5170.66 |
| -2620 | 60 | 5579.80 | 5519.80 | -2405.68 | 60 | 5154.46 | 5094.46 |
| -2564.57 | 76 | 5559.56 | 5483.56 | -2374.03 | 76 | 5182.67 | 5106.67 |
| -2539.5 | 92 | 5600.03 | 5508.03 | -2340.77 | 92 | 5207.64 | 5115.64 |
| -2490.91 | 108 | 5593.48 | 5485.48 | -2303.84 | 108 | 5225.28 | 5117.28 |
| -2466.31 | 124 | 5634.89 | 5510.89 | -2268.20 | 124 | 5245.49 | 5121.49 |
