# Supplementary material for: How to assess? Student preferences for methods to assess experiential learning: A best-worst scaling approach
Source: PLoS One. 2022 Oct 27;17(10):e0276745. doi: 10.1371/journal.pone.0276745 (PMC9612489; doi:10.1371/journal.pone.0276745)
Supplement: S4 Table — (DOCX) [file pone.0276745.s008.docx]

**S4 Table.** **Latent class model estimates and shares of preferences (SP) of assessment formats.**

| Assessment Format | Class 1 | | Class 2 | | Class 3 | | Class 4 | | Class 5 |
| --- | --- | --- | --- | --- | --- | --- | --- | --- | --- |
|  | Estimates | SP | Estimates | SP | Estimates | SP | Estimates | SP | SP |
| Final Project | 0.10 | 8.22*** | 2.04*** | 8.35*** | 1.33*** | 2.93*** | 2.18*** | 8.90*** | 6.33*** |
|  | (0.26) | (1.42) | (0.36) | (1.94) | (0.36) | (0.83) | (0.24) | (1.58) | (1.17) |
| Class participation | -1.07*** | 2.55*** | 2.43*** | 12.40*** | -0.12 | 0.69** | 0.50* | 1.66*** | 1.92*** |
|  | (0.26) | (0.55) | (0.38) | (2.61) | (0.36) | (0.23) | (0.24) | (0.36) | (0.43) |
| Homework assignments | -0.16 | 6.30*** | 1.92*** | 7.43*** | 0.82* | 1.77*** | -0.76*** | 0.47*** | 1.20*** |
|  | (0.27) | (1.13) | (0.39) | (1.75) | (0.36) | (0.52) | (0.23) | (0.11) | (0.27) |
| Analysis and discussion of case studies | 0.98*** | 19.78*** | 2.32*** | 11.06*** | 2.87*** | 13.70*** | 3.18*** | 24.18*** | 19.11*** |
|  | (0.29) | (3.35) | (0.39) | (2.45) | (0.41) | (3.31) | (0.28) | (3.68) | (3.37) |
| Written essay | -0.66* | 3.84*** | 1.98*** | 7.85*** | 1.18** | 2.52*** | 0.83*** | 2.29*** | 1.93*** |
|  | (0.29) | (0.73) | (0.37) | (1.85) | (0.37) | (0.71) | (0.23) | (0.46) | (0.44) |
| Portfolio | 0.56' | 12.99*** | 1.34*** | 4.18*** | 0.42 | 1.18** | 0.07 | 1.08*** | 14.62*** |
|  | (0.32) | (2.51) | (0.37) | (1.04) | (0.35) | (0.37) | (0.22) | (0.24) | (2.74) |
| Continuous quizzes of multiple choice questions | -0.47' | 4.64*** | 0.67' | 2.13*** | 2.79*** | 12.64*** | 0.43' | 1.55*** | 1.23*** |
|  | (0.25) | (0.89) | (0.35) | (0.56) | (0.42) | (3.10) | (0.23) | (0.33) | (0.26) |
| Continuous quizzes of open-ended questions | 0.68** | 14.61*** | 1.33*** | 4.13*** | 2.81*** | 12.85*** | 1.67*** | 5.34*** | 2.49*** |
|  | (0.26) | (2.63) | (0.35) | (1.00) | (0.42) | (3.05) | (0.24) | (0.95) | (0.51) |
| Open book exam | 0.22 | 9.24*** | 1.16*** | 3.48*** | 2.16*** | 6.74*** | 1.68*** | 5.39*** | 2.13*** |
|  | (0.27) | (1.57) | (0.35) | (0.85) | (0.38) | (1.76) | (0.24) | (0.96) | (0.47) |
| Professional presentations | -1.08*** | 2.52*** | 2.25*** | 10.30*** | 1.29*** | 2.82*** | 2.40*** | 11.02*** | 19.89*** |
|  | (0.29) | (0.50) | (0.37) | (2.31) | (0.37) | (0.80) | (0.25) | (1.95) | (4.06) |
| Peer evaluation | -1.42*** | 1.80*** | -0.21 | 0.88** | 2.51*** | 9.57*** | -1.22*** | 0.30*** | 3.07*** |
|  | (0.30) | (0.38) | (0.36) | (0.28) | (0.42) | (2.38) | (0.25) | (0.08) | (0.61) |
| Lab practices and simulations | -0.20 | 6.07*** | 3.20*** | 26.73*** | 3.71*** | 31.84*** | 3.60*** | 36.82*** | 25.64*** |
|  | (0.30) | (1.13) | (0.28) | (5.44) | (0.44) | (6.29) | (0.30) | (5.14) | (4.11) |
| Proctored exam |  | 7.43*** |  | 1.09** |  | 0.78** |  | 1.00*** | 0.44*** |
|  |  | (1.49) |  | (0.34) |  | (0.26) |  | (0.22) | (0.11) |
|  |  |  |  |  |  |  |  |  |  |
| Class share (%) | 17.92 |  | 12.26 |  | 12.26 |  | 33.02 |  |  |
| Class membership |  |  |  |  |  |  |  |  |  |
| Female | -0.64 |  | 0.05 |  | -1.65* |  | -0.78 |  |  |
|  | (0.72) |  | (0.86) |  | (0.81) |  | (0.59) |  |  |
| Year | -0.35 |  | -0.44' |  | -0.82** |  | -0.37' |  |  |
|  | (0.25) |  | (0.26) |  | (0.31) |  | (0.21) |  |  |
| First generation studying | 0.33 |  | 1.77* |  | 1.13 |  | -0.18 |  |  |
|  | (0.73) |  | (0.78) |  | (0.83) |  | (0.68) |  |  |
| Constant | 1.24 |  | 0.10 |  | 2.46* |  | 2.11* |  |  |
|  | (1.17) |  | (1.18) |  | (1.15) |  | (0.92) |  |  |

***Notes:*** Standard errors in parentheses. Levels of statistical significance:

' 0.1 * 0.05 ** 0.01 *** 0.001.
