# Supplementary material for: How to assess? Student preferences for methods to assess experiential learning: A best-worst scaling approach
Source: PLoS One. 2022 Oct 27;17(10):e0276745. doi: 10.1371/journal.pone.0276745 (PMC9612489; doi:10.1371/journal.pone.0276745)
Supplement: S5 Table — (DOCX) [file pone.0276745.s009.docx]

**S5 Table.** **Latent class model estimates and shares of preferences (SP) of assessment attributes.**

| Assessment Attribute | | Class 1 | | | Class 2 | | | | | | | | Class 3 | | | Class 4 |
| --- | --- | --- | --- | --- | --- | --- | --- | --- | --- | --- | --- | --- | --- | --- | --- | --- |
|  | Estimates | | SP | | Estimates | | | | SP | | | | Estimates | | SP | SP |
| Valid | | 0.70** | 6.01*** | | 3.03*** | | | | 5.68*** | | | | 1.25*** | | 1.94*** | 2.21*** |
|  | | (0.21) | (0.93) | | (0.32) | | | | (1.26) | | | | (0.33) | | (0.56) | (0.51) |
| Safe | | -0.10 | 2.71*** | | -0.08 | | | | 0.25** | | | | -0.62' | | 0.30** | 2.40*** |
|  | | (0.22) | (0.47) | | (0.30) | | | | (0.08) | | | | (0.32) | | (0.10) | (0.60) |
| Precise | | 1.17*** | 9.65*** | | 3.09*** | | | | 6.03*** | | | | 0.87** | | 1.33*** | 2.56*** |
|  | | (0.21) | (1.45) | | (0.32) | | | | (1.34) | | | | (0.30) | | (0.38) | (0.59) |
| Pertinent | | 1.89*** | 19.74*** | | 3.17*** | | | | 6.57*** | | | | 1.88*** | | 3.66*** | 8.05*** |
|  | | (0.24) | (2.85) | | (0.32) | | | | (1.47) | | | | (0.34) | | (1.00) | (1.73) |
| Simple | | 0.77*** | 6.43*** | | 0.80** | | | | 0.61*** | | | | 1.12*** | | 1.71*** | 0.23** |
|  | | (0.22) | (0.99) | | (0.27) | | | | (0.18) | | | | (0.31) | | (0.52) | (0.07) |
| Realistic | | 2.00*** | 22.09*** | | 3.94*** | | | | 14.18*** | | | | 3.97*** | | 29.63*** | 28.76*** |
|  | | (0.24) | (3.16) | | (0.34) | | | | (2.85) | | | | (0.39) | | (6.20) | (4.50) |
| Analytical | | 0.39' | 4.42*** | | 3.76*** | | | | 11.77*** | | | | 2.48*** | | 6.66*** | 14.68*** |
|  | | (0.21) | (0.70) | | (0.33) | | | | (2.16) | | | | (0.34) | | (1.54) | (2.71) |
| Promoter | | 0.20 | 3.63*** | | 3.36*** | | | | 7.90*** | | | | 2.64*** | | 7.84*** | 3.90*** |
|  | | (0.22) | (0.59) | | (0.33) | | | | (1.59) | | | | (0.33) | | (1.88) | (0.86) |
| Driving | | 1.43*** | 12.49*** | | 5.10*** | | | | 44.88*** | | | | 4.20*** | | 37.20*** | 34.66*** |
|  | | (0.22) | (1.83) | | (0.36) | | | | (5.89) | | | | (0.38) | | (6.51) | (5.10) |
| Strategic | | 0.29 | 3.98*** | | 0.92** | | | | 0.69*** | | | | -0.20 | | 0.46** | 0.04* |
|  | | (0.21) | (0.65) | | (0.29) | | | | (0.20) | | | | (0.29) | | (0.14) | (0.02) |
| Frequent | | 0.46* | 4.74*** | | 0.20 | | | | 0.33** | | | | 1.44*** | | 2.35** | 0.65*** |
|  | | (0.22) | (0.76) | | (0.31) | | | | (0.11) | | | | (0.34) | | (0.78) | (0.17) |
| Collective | | -0.97*** | 1.13*** | | 1.10*** | | | | 0.83*** | | | | 2.44*** | | 6.39*** | 1.78*** |
|  | | (0.24) | (0.23) | | (0.28) | | | | (0.23) | | | | (0.33) | | (1.62) | (0.51) |
| Fast | |  | 2.98*** | | | |  | | 0.28*** | | |  | | | 0.56*** | 0.07* |
|  | |  | (0.49) | | | |  | | (0.08) | | |  | | | (0.17) | (0.03) |
|  | |  |  | |  | | | |  | | | | |  |  |  |
| Class share | | 23.21 |  |  | | 30.36 | |  |  |  |  | | | 19.64 |  | 26.79 |
| Class membership | |  |  | |  | | | |  | | | | |  |  |  |
| Female | | 0.92 |  | | 0.59 | | | |  | | | | | -0.26 |  |  |
|  | | (0.66) |  | | (0.60) | | | |  | | | | | (0.63) |  |  |
| Year | | -0.30 |  | | -0.06 | | | |  | | | | | -0.08 |  |  |
|  | | (0.21) |  | | (0.20) | | | |  | | | | | (0.21) |  |  |
| First generation studying | | 0.42 |  | | -0.34 | | | |  | | | | | 0.13 |  |  |

***Notes:*** Standard errors in parentheses. Levels of statistical significance:

' 0.1, * 0.05, ** 0.01, and *** 0.001. Class 4 is the reference class.
