# Supplementary material for: How to assess? Student preferences for methods to assess experiential learning: A best-worst scaling approach
Source: PLoS One. 2022 Oct 27;17(10):e0276745. doi: 10.1371/journal.pone.0276745 (PMC9612489; doi:10.1371/journal.pone.0276745)
Supplement: S6 Table — (DOCX) [file pone.0276745.s010.docx]

**S6 Table.** **Pearson correlations between individual-specific B-W scores for assessment formats.**

| Assessment formats | | 1 | 2 | 3 | 4 | 5 | 6 | 7 | 8 | 9 | 10 | 11 | 12 | 13 |
| --- | --- | --- | --- | --- | --- | --- | --- | --- | --- | --- | --- | --- | --- | --- |
| 1 | Final Project | 1 |  |  |  |  |  |  |  |  |  |  |  |  |
| 2 | Participation in class | -0.06 | 1 |  |  |  |  |  |  |  |  |  |  |  |
| 3 | Homework assigments | -0.16 | -0.12 | 1 |  |  |  |  |  |  |  |  |  |  |
| 4 | Analysis and discussion of case studies | -0.13 | -0.01 | **-0.20** | 1 |  |  |  |  |  |  |  |  |  |
| 5 | Written essay | 0.11 | -0.09 | 0.03 | -0.04 | 1 |  |  |  |  |  |  |  |  |
| 6 | Portfolio | 0.04 | -0.12 | 0.13 | 0.00 | 0.02 | 1 |  |  |  |  |  |  |  |
| 7 | Continuous quizzes of multiple choice | **-0.25** | **-0.29** | 0.04 | **-0.19** | **-0.27** | **-0.28** | 1 |  |  |  |  |  |  |
| 8 | Continuous quizzes of open questions | -0.18 | **-0.22** | 0.01 | -0.09 | -0.08 | -0.18 | 0.09 | 1 |  |  |  |  |  |
| 9 | Open book exam | 0.11 | -0.11 | **-0.23** | **-0.26** | -0.15 | **-0.24** | 0.11 | **0.32** | 1 |  |  |  |  |
| 10 | Professional presentations | 0.06 | 0.15 | **-0.29** | 0.03 | -0.06 | -0.07 | -0.18 | **-0.39** | **-0.36** | 1 |  |  |  |
| 11 | Proctored exam | 0.00 | -0.13 | 0.01 | -0.11 | -0.11 | **-0.22** | 0.11 | **0.37** | **0.28** | **-0.38** | 1 |  |  |
| 12 | Peer evaluation | **-0.30** | -0.10 | -0.11 | -0.03 | **-0.21** | 0.05 | 0.05 | -0.16 | -0.03 | -0.03 | **-0.33** | 1 |  |
| 13 | Lab and simulations | -0.16 | 0.05 | **-0.24** | **0.21** | -0.01 | **-0.35** | -0.01 | -**0.27** | **-0.23** | **0.38** | **-0.29** | -0.04 | 1 |

***Note:*** Bolded values indicate statistical significance at the 0.05 level or lower.
