# Supplementary material for: How to assess? Student preferences for methods to assess experiential learning: A best-worst scaling approach
Source: PLoS One. 2022 Oct 27;17(10):e0276745. doi: 10.1371/journal.pone.0276745 (PMC9612489; doi:10.1371/journal.pone.0276745)
Supplement: S7 Table — (DOCX) [file pone.0276745.s011.docx]

**S7 Table.** **Pearson correlations between individual-specific B-W scores for assessment attributes.**

| Assessment attributes | | 1 | 2 | 3 | 4 | 5 | 6 | 7 | 8 | 9 | 10 | 11 | 12 | 13 |
| --- | --- | --- | --- | --- | --- | --- | --- | --- | --- | --- | --- | --- | --- | --- |
| 1 | Fast | 1 |  |  |  |  |  |  |  |  |  |  |  |  |
| 2 | Valid | -0.02 | 1 |  |  |  |  |  |  |  |  |  |  |  |
| 3 | Safe | **-0.22** | -0.10 | 1 |  |  |  |  |  |  |  |  |  |  |
| 4 | Precise | 0.13 | 0.16 | -0.12 | 1 |  |  |  |  |  |  |  |  |  |
| 5 | Pertinent | -0.16 | 0.18 | 0.14 | 0.17 | 1 |  |  |  |  |  |  |  |  |
| 6 | Simple | **0.30** | **-0.24** | **-0.21** | **0.28** | 0.04 | 1 |  |  |  |  |  |  |  |
| 7 | Realistic | 0.06 | **-0.42** | 0.10 | -0.15 | -0.07 | 0.00 | 1 |  |  |  |  |  |  |
| 8 | Analytical | **-0.21** | 0.00 | 0.02 | **-0.28** | **-0.22** | **-0.33** | -0.13 | 1 |  |  |  |  |  |
| 9 | Promoter | **-0.32** | -0.12 | -0.16 | **-0.22** | **-0.36** | **-0.23** | -0.05 | 0.10 | 1 |  |  |  |  |
| 10 | Driving | **-0.23** | 0.00 | **-0.19** | **-0.26** | **-0.27** | **-0.37** | -0.05 | **0.46** | **0.24** | 1 |  |  |  |
| 11 | Strategic | 0.12 | 0.06 | **-0.30** | 0.06 | -0.01 | 0.15 | -0.18 | **-0.35** | -0.09 | **-0.27** | 1 |  |  |
| 12 | Frequent | 0.01 | **-0.22** | 0.05 | **-0.24** | 0.00 | -0.06 | -0.14 | **-0.20** | **-0.20** | **-0.19** | -0.02 | 1 |  |
| 13 | Collective | **-0.32** | -0.12 | -0.12 | **-0.36** | **-0.40** | **-0.31** | 0.09 | 0.16 | **0.33** | **0.22** | **-0.28** | -0.02 | 1 |
| ***Note:*** Bolded values indicate statistical significance at the 0.05 level or lower. | | | | | | | | | | | | | | |
