# Supplementary material for: How to assess? Student preferences for methods to assess experiential learning: A best-worst scaling approach
Source: PLoS One. 2022 Oct 27;17(10):e0276745. doi: 10.1371/journal.pone.0276745 (PMC9612489; doi:10.1371/journal.pone.0276745)
Supplement: S8 Table — (DOCX) [file pone.0276745.s012.docx]

**S8 Table.** **Kendall's Tau correlations of personality traits with preferences for assessment formats.**

| Assessment Format | Neuroticism | Extroversion | Openness to experience | Agreeableness | Conscientiousness |
| --- | --- | --- | --- | --- | --- |
| Final Project | 0.04 | 0.01 | 0.01 | -0.01 | -0.05 |
| Participation in class | -0.06 | -0.06 | -0.03 | 0.07 | 0.06 |
| Homework assigments | -0.01 | -0.04 | -0.02 | -0.05 | **0.12** |
| Analysis and discussion of case studies | 0.00 | 0.03 | -0.03 | 0.07 | -0.07 |
| Written essay | 0.00 | -0.02 | 0.05 | -0.01 | -0.02 |
| Portfolio | -0.04 | 0.03 | -0.05 | 0.03 | 0.02 |
| Continuous quizzes of multiple choice | **0.06** | -0.02 | 0.04 | -0.05 | -0.03 |
| Continuous quizzes of open questions | 0.06 | -0.02 | -0.02 | -0.01 | -0.01 |
| Open book exam | 0.02 | 0.03 | -0.02 | -0.05 | 0.02 |
| Professional presentations | 0.01 | **0.06** | -0.02 | 0.01 | -0.04 |
| Proctored exam | 0.04 | 0.01 | 0.01 | -0.01 | -0.05 |
| Peer evaluation | -0.06 | **-0.06** | -0.03 | 0.07 | 0.06 |
| Lab and simulations | -0.01 | 0.02 | 0.04 | 0.04 | -0.08 |
| ***Note:*** Bolded values indicate statistical significance at the 0.05 level or lower. | | | | | |
