# Supplementary material for: How to assess? Student preferences for methods to assess experiential learning: A best-worst scaling approach
Source: PLoS One. 2022 Oct 27;17(10):e0276745. doi: 10.1371/journal.pone.0276745 (PMC9612489; doi:10.1371/journal.pone.0276745)
Supplement: S9 Table — (DOCX) [file pone.0276745.s013.docx]

**S9 Table.** **Kendall's Tau correlations of personality traits with preferences for assessment attributes.**

| Assessment Attribute | Neuroticism | Extroversion | Openness to experience | Agreeableness | Conscientiousness | |
| --- | --- | --- | --- | --- | --- | --- |
| Fast | 0.04 | **-0.06** | -0.02 | 0.00 | 0.05 | |
| Valid | 0.01 | 0.03 | -0.07 | 0.01 | 0.02 | |
| Safe | 0.01 | -0.03 | -0.05 | 0.06 | 0.02 | |
| Precise | 0.04 | 0.00 | -0.05 | 0.01 | 0.01 | |
| Pertinent | 0.04 | -0.03 | -0.04 | -0.03 | 0.05 | |
| Simple | 0.02 | **-0.06** | -0.07 | 0.01 | 0.10 | |
| Realistic | -0.04 | 0.02 | 0.02 | 0.01 | -0.01 | |
| Analytical | -0.06 | 0.00 | 0.00 | -0.03 | 0.09 | |
| Promoter | -0.03 | **0.06** | **0.08** | -0.03 | -0.07 | |
| Driving | -0.04 | -0.02 | **0.08** | 0.01 | -0.03 | |
| Strategic | 0.06 | -0.05 | 0.01 | -0.01 | -0.02 | |
| Frequent | 0.03 | 0.02 | 0.01 | -0.02 | -0.05 | |
| Collective | **-0.11** | **0.09** | **0.08** | 0.00 | -0.06 | |
| ***Note:*** Bolded values indicate statistical significance at the 0.05 level or lower. | | | | | |  |
