# Supplementary material for: How to assess? Student preferences for methods to assess experiential learning: A best-worst scaling approach
Source: PLoS One. 2022 Oct 27;17(10):e0276745. doi: 10.1371/journal.pone.0276745 (PMC9612489; doi:10.1371/journal.pone.0276745)
Supplement: S10 Table — (DOCX) [file pone.0276745.s014.docx]

**S10 Table.** **Kendall's Tau correlations of learning style with preferences for assessment formats.**

| Assessment Format | Active | Reflexive | Theoretical | Pragmatic |
| --- | --- | --- | --- | --- |
| Final Project | -0.04 | 0.06 | 0.00 | -0.02 |
| Participation in class | 0.04 | 0.00 | -0.02 | -0.02 |
| Homework assigments | -0.08 | -0.04 | 0.12 | 0.00 |
| Analysis and discussion of case studies | -0.04 | 0.00 | -0.01 | 0.05 |
| Written essay | -0.03 | **0.10** | -0.05 | -0.02 |
| Portfolio | -0.04 | 0.07 | 0.00 | -0.03 |
| Continuous quizzes of multiple choice | 0.02 | -0.04 | 0.02 | -0.01 |
| Continuous quizzes of open questions | -0.06 | 0.02 | -0.01 | 0.06 |
| Open book exam | -0.02 | 0.00 | -0.01 | 0.03 |
| Professional presentations | 0.07 | -0.02 | -0.04 | -0.01 |
| Proctored exam | 0.00 | **-0.10** | 0.06 | 0.04 |
| Peer evaluation | 0.08 | -0.06 | -0.04 | 0.01 |
| Lab and simulations | 0.05 | 0.02 | -0.03 | -0.05 |
| ***Note:*** Bolded values indicate statistical significance at the 0.05 level or lower. | | | | |
