# Supplementary material for: How to assess? Student preferences for methods to assess experiential learning: A best-worst scaling approach
Source: PLoS One. 2022 Oct 27;17(10):e0276745. doi: 10.1371/journal.pone.0276745 (PMC9612489; doi:10.1371/journal.pone.0276745)
Supplement: S11 Table — (DOCX) [file pone.0276745.s015.docx]

**S11 Table. Kendall's Tau correlations of learning style with preferences for assessment attributes.**

| Assessment Attribute | Active | Reflexive | Theoretical | Pragmatic |
| --- | --- | --- | --- | --- |
| Fast | -0.02 | -0.03 | 0.01 | 0.04 |
| Valid | 0.04 | 0.08 | -0.04 | -0.09 |
| Safe | 0.03 | -0.04 | -0.02 | 0.03 |
| Precise | -0.05 | 0.04 | 0.03 | **-0.02** |
| Pertinent | -0.03 | -0.03 | **0.15** | **-0.08** |
| Simple | -0.06 | -0.06 | **0.13** | 0.00 |
| Realistic | -0.02 | -0.07 | 0.03 | 0.05 |
| Analytical | **-0.08** | 0.05 | 0.04 | -0.01 |
| Promoter | 0.07 | 0.02 | -0.04 | -0.06 |
| Driving | 0.04 | 0.04 | **-0.11** | 0.03 |
| Strategic | -0.04 | **0.11** | -0.09 | 0.02 |
| Frequent | 0.06 | -0.04 | -0.02 | 0.00 |
| Collective | 0.02 | 0.01 | -0.07 | 0.03 |
| ***Note:*** Bolded values indicate statistical significance at the 0.05 level or lower. | | | | |
