# Supplementary material for: How to assess? Student preferences for methods to assess experiential learning: A best-worst scaling approach
Source: PLoS One. 2022 Oct 27;17(10):e0276745. doi: 10.1371/journal.pone.0276745 (PMC9612489; doi:10.1371/journal.pone.0276745)
Supplement: S1 File — (PDF) [file pone.0276745.s016.pdf]

<u>Encuesta: Atributos de métodos de evaluación</u>

¿Eres estudiante de pregrado de la Pontificia Universidad Católica de Chile?

( ) SI
( ) SI, estoy también postgrado
( ) NO

*Solo se prosigue, si marca la primera opción,

Aprendizaje y métodos de evaluación

A continuación, te presentaremos 13 escenarios de 4 atributos de métodos de evaluación cada uno. En cada escenario debes elegir el atributo que consideres **"Más importante"** y el atributo que consideres **"Menos importante"** para facilitar TU APRENDIZAJE.

Al contestar cada pregunta:

- Ten en mente que todos los atributos caracterizan a métodos de evaluación en cursos de ciencias aplicadas en tu universidad.
- Imagina que tú puedes escoger los atributos de métodos de evaluación que facilitan TU APRENDIZAJE.
- Elige basado en lo que realmente piensas, no en lo que crees que es más común o factible en tu carrera.

Ejemplo:

Considerando solo estos 4 atributos de métodos de evaluación, indica:
¿Cuál es el “Más importante" y cuál es el "Menos importante" para facilitar tu aprendizaje?

Juan cree que el atributo RÁPIDO es el “Más importante" y el atributo SENCILLO es el “Menos importante” para facilitar su aprendizaje. Por lo tanto, en la siguiente tabla, el marcó:

Más importante	ATRIBUTO DE MÉTODOS DE EVALUACIÓN PARA FACILITAR APRENDIZAJE	Menos importante
X	**Rápido**<br>Implica poco tiempo en la realización y preparación.	
	**Colectivo**<br>Actividades y evaluaciones realizadas en grupo.	
	**Sencillo**<br>Es de fácil realización y la tarea/actividad/pregunta es familiar para el estudiante.	X
	**Impulsor**<br>Desarrolla diferentes tipos de habilidades tales como pensamiento creativo, pensamiento crítico, resolución de problemas, etc.	

1. **Considerando solo estos 4 atributos de métodos de evaluación, indica:**
   ¿Cuál es el “Más importante" y cuál es el "Menos importante" para facilitar tu aprendizaje?

Más importante	ATRIBUTO DE MÉTODOS DE EVALUACIÓN PARA FACILITAR APRENDIZAJE	Menos importante
	**Válido**<br>Apropiado para evaluar el logro de los objetivos de aprendizaje.	
	**Sencillo**<br>Es de fácil realización y la tarea/actividad/pregunta es familiar para el estudiante.	
	**Realista**<br>Desarrolla habilidades profesionales transferibles al mundo real.	
	**Colectivo**<br>Actividades y evaluaciones realizadas en grupo.	

2. **Considerando solo estos 4 atributos de métodos de evaluación, indica:**
   ¿Cuál es el “Más importante" y cuál es el "Menos importante" para facilitar tu aprendizaje?

Más importante	ATRIBUTO DE MÉTODOS DE EVALUACIÓN PARA FACILITAR APRENDIZAJE	Menos importante
	**Válido**<br>Apropiado para evaluar el logro de los objetivos de aprendizaje.	
	**Pertinente**<br>Refleja el nivel real de conocimiento del estudiante.	
	**Analítico**<br>Promueve análisis, discusión y debate.	
	**Impulsor**<br>Desarrolla diferentes tipos de habilidades tales como pensamiento creativo, pensamiento crítico, resolución de problemas, etc.	

3. **Considerando solo estos 4 atributos de métodos de evaluación, indica:**
   ¿Cuál es el “Más importante" y cuál es el "Menos importante" para facilitar tu aprendizaje?

Más importante	ATRIBUTO DE MÉTODOS DE EVALUACIÓN PARA FACILITAR APRENDIZAJE	Menos importante
	**Válido**<br>Apropiado para evaluar el logro de los objetivos de aprendizaje.	
	**Seguro**<br>Su diseño considera medidas preventivas para que la evaluación esté libre de trampas, fraude y adivinar las respuestas.	
	**Preciso**<br>De fácil entendimiento, poca ambigüedad.	
	**Promotor**<br>Promueve participación activa del estudiante.	

4. **Considerando solo estos 4 atributos de métodos de evaluación, indica:**
   ¿Cuál es el “Más importante" y cuál es el "Menos importante" para facilitar tu aprendizaje?

Más importante	ATRIBUTO DE MÉTODOS DE EVALUACIÓN PARA FACILITAR APRENDIZAJE	Menos importante
	**Seguro**	

	Su diseño considera medidas preventivas para que la evaluación esté libre de trampas, fraude y adivinar las respuestas.	
	**Realista**<br>Desarrolla habilidades profesionales transferibles al mundo real.	
	**Analítico**<br>Promueve análisis, discusión y debate.	
	**Estratégico**<br>Alta probabilidad de obtener buenas calificaciones.	

5. **Considerando solo estos 4 atributos de métodos de evaluación, indica: ¿Cuál es el “Más importante" y cuál es el "Menos importante" para facilitar tu aprendizaje?**

Más importante	ATRIBUTO DE MÉTODOS DE EVALUACIÓN PARA FACILITAR APRENDIZAJE	Menos importante
	**Rápido**<br>Implica poco tiempo en la realización y preparación.	
	**Preciso**<br>De fácil entendimiento, poca ambigüedad.	
	**Pertinente**<br>Refleja el nivel real de conocimiento del estudiante.	
	**Realista**<br>Desarrolla habilidades profesionales transferibles al mundo real.	

6. **Considerando solo estos 4 atributos de métodos de evaluación, indica: ¿Cuál es el “Más importante" y cuál es el "Menos importante" para facilitar tu aprendizaje?**

Más importante	ATRIBUTO DE MÉTODOS DE EVALUACIÓN PARA FACILITAR APRENDIZAJE	Menos importante
	**Rápido**<br>Implica poco tiempo en la realización y preparación.	
	**Válido**<br>Apropiado para evaluar el logro de los objetivos de aprendizaje.	
	**Estratégico**<br>Alta probabilidad de obtener buenas calificaciones.	
	**Frecuente**<br>Se realiza repetidas veces durante el semestre.	

7. **Considerando solo estos 4 atributos de métodos de evaluación, indica: ¿Cuál es el “Más importante" y cuál es el "Menos importante" para facilitar tu aprendizaje?**

Más importante	ATRIBUTO DE MÉTODOS DE EVALUACIÓN PARA FACILITAR APRENDIZAJE	Menos importante
	**Preciso**<br>De fácil entendimiento, poca ambigüedad..	
	**Impulsor**<br>Desarrolla diferentes tipos de habilidades tales como pensamiento creativo, pensamiento crítico, resolución de problemas, etc.	
	**Estratégico**<br>Alta probabilidad de obtener buenas calificaciones.	
	**Colectivo**<br>Actividades y evaluaciones realizadas en grupo.	

8. **Considerando solo estos 4 atributos de métodos de evaluación, indica: ¿Cuál es el "Más importante" y cuál es el "Menos importante" para facilitar tu aprendizaje?**

Más importante	ATRIBUTO DE MÉTODOS DE EVALUACIÓN PARA FACILITAR APRENDIZAJE	Menos importante
	**Preciso**<br>De fácil entendimiento, poca ambigüedad.	
	**Sencillo**<br>Es de fácil realización y la tarea/actividad/pregunta es familiar para el estudiante.	
	**Analítico**<br>Promueve análisis, discusión y debate.	
	**Frecuente**<br>Se realiza repetidas veces durante el semestre.	

9. **Considerando solo estos 4 atributos de métodos de evaluación, indica: ¿Cuál es el "Más importante" y cuál es el "Menos importante" para facilitar tu aprendizaje?**

Más importante	ATRIBUTO DE MÉTODOS DE EVALUACIÓN PARA FACILITAR APRENDIZAJE	Menos importante
	**Rápido**<br>Implica poco tiempo en la realización y preparación.	
	**Analítico**<br>Promueve análisis, discusión y debate.	
	**Promotor**<br>Promueve participación activa del estudiante.	
	**Colectivo**<br>Actividades y evaluaciones realizadas en grupo.	

10. **Considerando solo estos 4 atributos de métodos de evaluación, indica: ¿Cuál es el "Más importante" y cuál es el "Menos importante" para facilitar tu aprendizaje?**

Más importante	ATRIBUTO DE MÉTODOS DE EVALUACIÓN PARA FACILITAR APRENDIZAJE	Menos importante
	**Rápido**<br>Implica poco tiempo en la realización y preparación.	
	**Seguro**<br>Su diseño considera medidas preventivas para que la evaluación esté libre de trampas, fraude y adivinar las respuestas.	
	**Sencillo**<br>Es de fácil realización y la tarea/actividad/pregunta es familiar para el estudiante.	
	**Impulsor**<br>Desarrolla diferentes tipos de habilidades tales como pensamiento creativo, pensamiento crítico, resolución de problemas, etc.	

11. **Considerando solo estos 4 atributos de métodos de evaluación, indica: ¿Cuál es el "Más importante" y cuál es el "Menos importante" para facilitar tu aprendizaje?**

Más importante	ATRIBUTO DE MÉTODOS DE EVALUACIÓN PARA FACILITAR APRENDIZAJE	Menos importante
	**Seguro**<br>Su diseño considera medidas preventivas para que la evaluación esté libre de trampas, fraude y	

	adivinar las respuestas..	
	**Pertinente**<br>Refleja el nivel real de conocimiento del estudiante.	
	**Frecuente**<br>Se realiza repetidas veces durante el semestre.	
	**Colectivo**<br>Actividades y evaluaciones realizadas en grupo.	

12. Considerando solo estos 4 atributos de métodos de evaluación, indica: ¿Cuál es el "Más importante" y cuál es el "Menos importante" para facilitar tu aprendizaje?

Más importante	ATRIBUTO DE MÉTODOS DE EVALUACIÓN PARA FACILITAR APRENDIZAJE	Menos importante
	**Pertinente**<br>Refleja el nivel real de conocimiento del estudiante.	
	**Sencillo**<br>Es de fácil realización y la tarea/actividad/pregunta es familiar para el estudiante.	
	**Promotor**<br>Promueve participación activa del estudiante.	
	**Estratégico**<br>Alta probabilidad de obtener buenas calificaciones.	

13. Considerando solo estos 4 atributos de métodos de evaluación, indica: ¿Cuál es el "Más importante" y cuál es el "Menos importante" para facilitar tu aprendizaje?

Más importante	ATRIBUTO DE MÉTODOS DE EVALUACIÓN PARA FACILITAR APRENDIZAJE	Menos importante
	**Realista**<br>Desarrolla habilidades profesionales transferibles al mundo real.	
	**Promotor**<br>Promueve participación activa del estudiante.	
	**Impulsor**<br>Desarrolla diferentes tipos de habilidades tales como pensamiento creativo, pensamiento crítico, resolución de problemas, etc.	
	**Frecuente**<br>Se realiza repetidas veces durante el semestre.	

Ahora queremos conocer tu opinión considerando todos las opciones

14. **¿Cuál de todos los 13 atributos de métodos de evaluación consideras que son “Muy importantes” para facilitar tu aprendizaje? Escoge todos los atributos que son “Muy importantes”.**

Considero que los siguientes atributos son “muy importantes” para mi aprendizaje:

ATRIBUTOS DE MÉTODOS DE EVALUACIÓN	
**Rápido**<br>Implica poco tiempo en la realización y preparación.	
**Válido**<br>Apropiado para evaluar el logro de los objetivos de aprendizaje.	
**Seguro**<br>Su diseño considera medidas preventivas para que la evaluación esté libre de trampas, fraude y adivinar las respuestas.	
**Preciso**<br>De fácil entendimiento, poca ambigüedad.	
**Pertinente**<br>Refleja el nivel real de conocimiento del estudiante.	
**Sencillo**<br>Es de fácil realización y la tarea/actividad/pregunta es familiar para el estudiante.	
**Realista**<br>Desarrolla habilidades profesionales transferibles al mundo real.	
**Analítico**<br>Promueve análisis, discusión y debate.	
**Promotor**<br>Promueve participación activa del estudiante.	
**Impulsor**<br>Desarrolla diferentes tipos de habilidades tales como pensamiento creativo, pensamiento crítico, resolución de problemas, etc.	
**Estratégico**<br>Alta probabilidad de obtener buenas calificaciones.	
**Frecuente**<br>Se realiza repetidas veces durante el semestre.	
**Colectivo**<br>Actividades y evaluaciones realizadas en grupo.	

Considero que ninguno de los 13 atributos son “muy importantes” para mi aprendizaje	

Ahora queremos conocer tu preferencia considerando todos las opciones

15. **¿Cuál de todos los 13 atributos de métodos de evaluación son los que “te gustan mucho”, independientemente si facilita tu aprendizaje o no? Escoge todos los atributos que “te gustan mucho”.**

Considero que los siguientes atributos “me gustan mucho”, independientemente si creo que facilitan mi aprendizaje o no:

ATRIBUTOS DE MÉTODOS DE EVALUACIÓN	
**Rápido**<br>Implica poco tiempo en la realización y preparación.	
**Válido**<br>Apropiado para evaluar el logro de los objetivos de aprendizaje.	
**Seguro**<br>Su diseño considera medidas preventivas para que la evaluación esté libre de trampas, fraude y adivinar las respuestas.	
**Preciso**<br>De fácil entendimiento, poca ambigüedad.	
**Pertinente**<br>Refleja el nivel real de conocimiento del estudiante.	
**Sencillo**<br>Es de fácil realización y la tarea/actividad/pregunta es familiar para el estudiante.	
**Realista**<br>Desarrolla habilidades profesionales transferibles al mundo real.	
**Analítico**<br>Promueve análisis, discusión y debate.	
**Promotor**<br>Promueve participación activa del estudiante.	
**Impulsor**<br>Desarrolla diferentes tipos de habilidades tales como pensamiento creativo, pensamiento crítico, resolución de problemas, etc.	
**Estratégico**<br>Alta probabilidad de obtener buenas calificaciones.	
**Frecuente**<br>Se realiza repetidas veces durante el semestre.	
**Colectivo**<br>Actividades y evaluaciones realizadas en grupo.	

Considero que ninguno de los 13 atributos “Me gustan mucho”, independientemente si creo que facilita mi aprendizaje o no.	

Imagina que puedes diseñar tu método de evaluación ideal

16. **¿Cuál es la probabilidad con la cual desearías que cada atributo aparezca en tu método de evaluación ideal**?
(Puedes usar un rango de 0% al 100%, recuerda que el total debe sumar 100%)

Ejemplo:

Juan desearía que su método de evaluación sea REALISTA (enfocado en desarrollar habilidades profesionales transferibles al mundo real) el 30% de las veces. Por otro lado, Juan considera que su método de evaluación debe ser SEGURO (enfocado en tener medidas preventivas para que la evaluación esté libre de trampas, fraude y adivinar las respuestas) el 3% de las veces. y asi sucesivamente.

Por tanto, Juan indica lo siguiente:

Seguro_______3%
Realista______30%
……….................
……………………

TOTAL 100%

Yo creo que mi método de evaluación ideal debería presentar los siguientes atributos con un % de probabilidad:

ATRIBUTOS DE MÉTODOS DE EVALUACIÓN	0-100%
**Rápido**<br>Implica poco tiempo en la realización y preparación.	
**Válido**<br>Apropiado para evaluar el logro de los objetivos de aprendizaje.	
**Seguro**<br>Su diseño considera medidas preventivas para que la evaluación esté libre de trampas, fraude y adivinar las respuestas.	
**Preciso**<br>De fácil entendimiento, poca ambigüedad.	
**Pertinente**<br>Refleja el nivel real de conocimiento del estudiante.	
**Sencillo**<br>Es de fácil realización y la tarea/actividad/pregunta es familiar para el estudiante.	
**Realista**<br>Desarrolla habilidades profesionales transferibles al mundo real.	
**Analítico**<br>Promueve análisis, discusión y debate.	
**Promotor**<br>Promueve participación activa del estudiante.	
**Impulsor**<br>Desarrolla diferentes tipos de habilidades tales como pensamiento creativo, pensamiento crítico, resolución de problemas, etc.	
**Estratégico**<br>Alta probabilidad de obtener buenas calificaciones.	
**Frecuente**<br>Se realiza repetidas veces durante el semestre.	
**Colectivo**<br>Actividades y evaluaciones realizadas en grupo.	
**Total**	**100%**

Estilos de Aprendizaje

Ahora, te describiremos algunas definiciones. Por favor lee cuidadosamente y escoge la opción con la que más te identificas.

Considerando las siguientes definiciones de **estilos de aprendizaje:**

Estilo de Aprendizaje	Descripción
**ESTILO A**	Busca experiencias nuevas, son de mente abierta, nada escépticos y realizan con entusiasmo tareas nuevas. *Características: animador, improvisador, arriesgado y espontáneo.*

ESTILO B

Antepone la reflexión a la acción, observa con detenimiento las distintas experiencias. *Características: ponderado, concienzudo, receptivo, analítico y exhaustivo.*

ESTILO C

Necesita integrar la experiencia en un marco teórico de referencia. Busca la racionalidad y la objetividad huyendo de lo subjetivo y lo ambiguo.
Características: metódico, lógico, objetivo, crítico y Estructurado.

ESTILO D Le gusta actuar rápidamente y con seguridad con aquellas ideas y proyectos que les atraen.
Características: experimentador, práctico, directo y eficaz.

---

17. ¿Con qué estilo de aprendizaje te identificas más?

( ) Estilo A *Características: animador, improvisador, arriesgado y espontáneo.*
( ) Estilo B *Características: ponderado, concienzudo, receptivo, analítico y exhaustivo.*
( ) Estilo C *Características: metódico, lógico, objetivo, crítico y estructurado.*
( ) Estilo D *Características: experimentador, práctico, directo y eficaz.*

18. ¿Con qué estilo de aprendizaje te identificas menos?

( ) Estilo A *Características: animador, improvisador, arriesgado y espontáneo.*
( ) Estilo B *Características: ponderado, concienzudo, receptivo, analítico y exhaustivo.*
( ) Estilo C *Características: metódico, lógico, objetivo, crítico y estructurado.*
( ) Estilo D *Características: experimentador, práctico, directo y eficaz.*

Rasgos de personalidad

Considerando las siguientes definiciones de **rasgos de personalidad:**

Rasgos de personalidad	Descripción
**TIPO A**	Refiere al ajuste emocional, la tendencia a experimentar emociones negativas, baja tolerancia a la frustración, así como a comportamientos impulsivos. *Cualidades: ansiedad, hostilidad, depresión, timidez, impulsividad, y vulnerabilidad.*

TIPO B

Refiere a la cantidad y calidad de interacciones interpersonales. Se compone de sociabilidad, alto nivel de actividad, asertividad y búsqueda de sensaciones.
Cualidades: afecto, asertividad, actividad, búsqueda de emociones y Emociones positivas.

TIPO C Refiere a la búsqueda de experiencias nuevas, originalidad, tendencia a la imaginación y atrevimiento.
Cualidades: fantasía, estética, sentimientos, acciones, ideas y valores.

TIPO D Refiere a una actitud cooperativa, empática y comprometida con otras personas.
Cualidades: franqueza, altruismo, modestia, confianza, actitud conciliadora y sensibilidad interpersonal.

TIPO E Refleja el grado de organización, persistencia, control y conducta dirigida a metas.
Cualidades son competencia, orden, necesidad de logro, sentido del deber, deliberación, y autodisclipina.

---

19. ¿Con qué tipo de personalidad te identificas más?

( ) Tipo A *Cualidades: ansiedad, hostilidad, depresión, timidez, impulsividad, y vulnerabilidad.*
( ) Tipo B *Cualidades: afecto, asertividad, actividad, búsqueda de emociones y Emociones positivas.*
( ) Tipo C *Cualidades: fantasía, estética, sentimientos, acciones, ideas y valores.*
( ) Tipo D *Cualidades: franqueza, altruismo, modestia, confianza, actitud conciliadora y sensibilidad interpersonal.*

( ) Tipo E *Cualidades: competencia, orden, necesidad de logro, sentido del deber, deliberación, y autodisclipina.*

20. ¿Con qué tipo de personalidad te identificas menos?

( ) Tipo A *Cualidades: ansiedad, hostilidad, depresión, timidez, impulsividad, y vulnerabilidad.*
( ) Tipo B *Cualidades: afecto, asertividad, actividad, búsqueda de emociones y Emociones positivas.*
( ) Tipo C *Cualidades: fantasía, estética, sentimientos, acciones, ideas y valores.*
( ) Tipo D *Cualidades: franqueza, altruismo, modestia, confianza, actitud conciliadora y sensibilidad interpersonal.*
( ) Tipo E *Cualidades: competencia, orden, necesidad de logro, sentido del deber, deliberación, y autodisclipina.*

Considerando el contexto de confinamiento actual:

21. **¿Sientes que tienes que estudiar más para obtener buenas calificaciones en tus evaluaciones realizadas en clases en línea ?**
    ( ) Si
    ( ) No

22. **Los métodos de evaluación usados en tus clases en línea ¿Perjudicaron o beneficiaron tus calificaciones académicas?**
    ( ) Perjudicaron mucho mis calificaciones académicas.
    ( ) Perjudicaron poco mis calificaciones académicas.
    ( ) Ni perjudicaron ni beneficiaron mis calificaciones académicas.
    ( ) Beneficiaron poco mis calificaciones académicas.
    ( ) Beneficiaron mucho mis calificaciones académicas.

23. **¿Qué factor ha afectado tu desempeño durante las evaluaciones en línea desde el inicio de la pandemia? (Seleccione todas las que corresponda).**
    ( ) Falta de interacción con el profesor.
    ( ) Escasa participación por parte de tus compañeros.
    ( ) Capacidad de atención limitada.
    ( ) Falta de recursos (tecnológicos, físicos, entre otros).
    ( ) Carente preparación por parte de los docentes para educación en línea.
    ( ) Otros. Detallar: ___
    ( ) No he presentado ningún problema.

24. **¿Consideras que tu preferencia por los métodos de evaluación han cambiado a raíz de tu experiencia con clases en línea?**
    ( ) Si, ahora prefiero evaluaciones en línea.
    ( ) Si, ahora prefiero más evaluaciones presenciales.
    ( ) Si, ahora prefiero un sistema de evaluación que combine evaluaciones presenciales y en línea.
    ( ) No, siempre he preferido evaluaciones en línea.
    ( ) No, siempre he preferido evaluaciones presenciales.
    ( ) No, siempre he preferido un sistema de evaluación que combine evaluaciones presenciales y en línea.

Gracias por llegar tan lejos! Casi has terminado, lo prometemos. Solo quedan pocas preguntas más sobre ti.

25. **¿Consideras que la educación en línea (incluyendo evaluaciones en línea) es más eficiente (optimiza recursos y tiempo) para lograr los objetivos de aprendizaje tanto como la educación presencial?**
    ( ) Es mucho más eficiente
    ( ) Es eficiente
    ( ) Ni eficiente ni deficiente
    ( ) Es deficiente
    ( ) Es muy deficiente

26. **¿Cómo consideras que es el acceso a internet en tu hogar para el desarrollo de sus evaluaciones académicas?**

( ) Muy Bueno
( ) Bueno
( ) Regular
( ) Malo
( ) Muy malo

27. **¿Tú o tu familia recibe ayuda financiera (diferente a cualquier apoyo financiero para tus estudios como becas o créditos) por parte del estado/gobierno?**
( ) Si
( ) No
( ) No sé

28. **¿Cuál es la carrera profesional que estás estudiando?**
(_________)

29. **¿En qué año académico te encuentras?**
( ) Primer año
( ) Segundo año
( ) Tercer año
( ) Cuarto año
( ) Quinto año
( ) Sexto año
( ) Séptimo año

30. **¿Te identificas con alguno de estos grupos? Puedes marcar más de una opción.**
( ) De primera generación (estudiantes que son primeros en sus familias en asistir a la universidad).
( ) Perteneciente a un hogar con bajos ingresos.
( ) Perteneciente a una etnia u pueblo originario (Mapuche, Aymara, Diaguita, entre otros).
( ) Estudiantes de bajo rendimiento académico.
( ) Proviene de una zona rural.
( ) LGTBIQ+ (lesbiana, gay, bisexual, transgénero, transexual, trasgenero, intersexual y otros).
( ) Estudiantes con algún tipo de discapacidad.
( ) Ninguno

31. **Durante el periodo de tus clases en línea (en pandemia) ¿Has sentido que perteneces a un aula inclusiva? (aula que integra el aprendizaje y participación de todos los alumnos pese a sus diferencias raciales, sexuales, culturales, género, entre otros).**
( ) Mucho
( ) Bastante
( ) Regular
( ) Poco
( ) Nada

32. **Considerando las clases presenciales que tenías antes de la pandemia, ¿Sientes que perteneciste a un aula inclusiva? (aula que integra el aprendizaje y participación de todos los alumnos pese a sus diferencias raciales, sexuales, culturales, género, entre otros).**
( ) Mucho
( ) Bastante

( ) Regular
( ) Poco
( ) Nada

33. ¿Qué edad tienes?
(___) años

34. ¿Con qué género te identificas?
( ) Masculino
( ) Femenino
( ) Prefiero no decir
( ) Otro:___

35. ¿Cuántos días hay en una semana?
( ) 1
( ) 2
( ) 3
( ) 4
( ) 5
( ) 6
( ) 7

36. Ingresa tu correo para que entres en el sorteo de la gift card: ______________________________

Encuesta: Métodos de evaluación

¿Eres estudiante de pregrado de la Pontificia Universidad Católica de Chile?

( ) SI
( ) SI, estoy también postgrado
( ) NO

*Solo se prosigue, si marca la primera opción

Aprendizaje y métodos de evaluación

A continuación, te presentaremos 13 escenarios de 4 métodos de evaluación cada uno. En cada escenario debes elegir el método de evaluación que consideres **"Más importante"** y el método de evaluación que consideres **"Menos importante"** para facilitar TU APRENDIZAJE.

Al contestar cada pregunta:

- Imagina que tú puedes escoger los métodos de evaluación que facilitan TU APRENDIZAJE.
- Elige basado en lo que realmente piensas, no en lo que crees que es más común o factible en tu carrera.

  Ejemplo:

  Considerando solo estos 4 métodos de evaluación, indica:
  ¿Cuál es el "Más importante" y cuál es el "Menos importante" para facilitar tu aprendizaje?

  Juan cree que el PORTAFOLIO es el método de evaluación "Más importante" y PRUEBAS CONTINUAS DE OPCIÓN MÚLTIPLE es el método de evaluación "Menos importante" para facilitar su aprendizaje. Por lo tanto, en la siguiente tabla, el marcó:

**Más importante**	METODO DE EVALUACION PARA FACILITAR APRENDIZAJE	**Menos importante**
**X**	**Portafolio**<br>Es una compilación de trabajos que el estudiante entrega de forma recurrente.<br>Su evaluación puede ser continua durante el semestre o al final del semestre.<br>Puede incluir trabajos como: documentos escritos, trabajos manuales, registros audiovisuales, entre otros.<br>Su realización es *individual.*	
	**Ensayo escrito**<br>Se evalúa el conocimiento sobre un tema específico y la opinión del estudiante.<br>Se evalúa el uso de la información (teoría) para emitir juicios.<br>Su realización es *individual.*	
	**Pruebas continuas de opción múltiple**<br>Constan de preguntas las cuales tienen un número limitado de opciones de respuestas.<br>Cada pregunta evalúa una única habilidad y/o contenido.<br>Su realización es *individual.*	**X**
	**Exámenes de libro abierto**<br>Contienen preguntas de análisis que no se pueden encontrar fácilmente en ninguna fuente (internet y libros).<br>Mide el conocimiento profundo a través de la aplicación de lo aprendido.	

1. **Considerando solo estos 4 métodos de evaluación, indica:**
¿Cuál es el “Más importante" y cuál es el "Menos importante" para facilitar tu aprendizaje?

Más importante	MÉTODO DE EVALUACIÓN PARA FACILITAR APRENDIZAJE	Menos importante
	**Participación en clases**<br>Se considera la intervención del estudiante durante el desarrollo de la clase.<br>Su evaluación es continua durante el semestre.<br>Su evaluación es *individual*.	
	**Portafolio**<br>Es una compilación de trabajos que el estudiante entrega de forma recurrente.<br>Su evaluación puede ser continua durante el semestre o al final del semestre.<br>Puede incluir trabajos como: documentos escritos, trabajos manuales y registros audiovisuales.<br>Su realización es *individual*.	
	**Pruebas continuas de opción múltiple**<br>Constan de preguntas las cuales tienen un número limitado de opciones de respuestas.<br>Cada pregunta evalúa una única habilidad y/o contenido.<br>Su realización es *individual*.	
	**Talleres, laboratorios y simulaciones**<br>Son evaluaciones basadas en actividades prácticas.<br>Permite que alumnos ensayen habilidades técnicas.<br>Su realización es *individual o grupal*.	

2. **Considerando solo estos 4 métodos de evaluación, indica:**
¿Cuál es el “Más importante" y cuál es el "Menos importante" para facilitar tu aprendizaje?

Más importante	MÉTODO DE EVALUACIÓN PARA FACILITAR APRENDIZAJE	Menos importante
	**Participación en clases**<br>Se considera la intervención del estudiante durante el desarrollo de la clase.<br>Su evaluación es continua durante el semestre.<br>Su evaluación es *individual*.	
	**Ensayo escrito**<br>Se evalúa el conocimiento sobre un tema específico y la opinión del estudiante.<br>Se evalúa el uso de la información (teoría) para emitir juicios.<br>Su realización es *individual*.	
	**Pruebas continuas de preguntas abiertas**<br>Pueden realizarse en modalidad escrita u oral.<br>Cada pregunta evalúa más de una habilidad o contenido.<br>Miden el conocimiento basados en las propias respuestas del estudiante.<br>Su realización es *individual*.	
	**Presentaciones o demostraciones profesionales**<br>Se realizan sobre un tema en específico usando materiales audiovisuales.<br>Se evalúa el conocimiento de un tema y habilidades comunicativas.<br>Su realización es *individual o grupal*.	

3. **Considerando solo estos 4 métodos de evaluación, indica:**
¿Cuál es el "Más importante" y cuál es el "Menos importante" para facilitar tu aprendizaje?

Más importante	MÉTODO DE EVALUACIÓN PARA FACILITAR APRENDIZAJE	Menos importante
	**Participación en clases**<br>Se considera la intervención del estudiante durante el desarrollo de la clase.<br>Su evaluación es continua durante el semestre.<br>Su evaluación es *individual*.	
	**Tareas en casa**<br>Se evalúa el trabajo realizado fuera del horario de clases.<br>Los plazos de entrega suelen ser cortos.<br>Su evaluación es continua durante el semestre.<br>Su realización es generalmente *individual*.	
	**Análisis y discusiones de casos reales**<br>Busca reflejar lo aprendido basado en análisis de casos de estudio.<br>El análisis se ve materializado en un documento escrito o exposición oral.<br>Para su evaluación se considera el dominio del tema, buena redacción y coherencia.<br>Su realización es *individual o grupal*.	
	**Exámenes de libro abierto**<br>Contienen preguntas de análisis que no se pueden encontrar fácilmente en ninguna fuente (internet y libros).<br>Mide el conocimiento profundo a través de la aplicación de lo aprendido.	

4. **Considerando solo estos 4 métodos de evaluación, indica:**
¿Cuál es el "Más importante" y cuál es el "Menos importante" para facilitar tu aprendizaje?

Más importante	MÉTODO DE EVALUACIÓN PARA FACILITAR APRENDIZAJE	Menos importante
	**Tareas en casa**<br>Se evalúa el trabajo realizado fuera del horario de clases.<br>Los plazos de entrega suelen ser cortos.<br>Su evaluación es continua durante el semestre.<br>Su realización es generalmente *individual*.	
	**Pruebas continuas de opción múltiple**<br>Constan de preguntas las cuales tienen un número limitado de opciones de respuestas.<br>Cada pregunta evalúa una única habilidad y/o contenido.<br>Su realización es *individual*.	
	**Pruebas continuas de preguntas abiertas**<br>Pueden realizarse en modalidad escrita u oral.<br>Cada pregunta evalúa más de una habilidad o contenido.<br>Miden el conocimiento basados en las propias respuestas del estudiante.<br>Su realización es *individual*.	
	**Examen escrito supervisado**<br>Es de tiempo limitado y supervisado.<br>Se evalúa de forma remota mediante el uso de cámaras web para rastrear la actividad de los estudiantes durante el examen.<br>Su realización es *individual*.	

5. **Considerando solo estos 4 métodos de evaluación, indica:**
¿Cuál es el “Más importante" y cuál es el "Menos importante" para facilitar tu aprendizaje?

Más importante	MÉTODO DE EVALUACIÓN PARA FACILITAR APRENDIZAJE	Menos importante
	**Proyecto final**<br>Implica considerable análisis y dedicación por parte del estudiante.<br>Las instrucciones son entregadas generalmente al inicio del semestre.<br>Tiene mucho peso en la calificación final.<br>Su realización es *individual o grupal*.	
	**Análisis y discusiones de casos reales**<br>Busca reflejar lo aprendido basado en análisis de casos de estudio.<br>El análisis se ve materializado en un documento escrito o exposición oral.<br>Para su evaluación se considera el dominio del tema, buena redacción y coherencia.<br>Su realización es *individual o grupal*.	
	**Ensayo escrito**<br>Se evalúa el conocimiento sobre un tema específico y la opinión del estudiante.<br>Se evalúa el uso de la información (teoría) para emitir juicios.<br>Su realización es *individual*.	
	**Pruebas continuas de opción múltiple**<br>Constan de preguntas las cuales tienen un número limitado de opciones de respuestas.<br>Cada pregunta evalúa una única habilidad y/o contenido.<br>Su realización es *individual*.	

6. **Considerando solo estos 4 métodos de evaluación, indica:**
¿Cuál es el “Más importante" y cuál es el "Menos importante" para facilitar tu aprendizaje?

Más importante	MÉTODO DE EVALUACIÓN PARA FACILITAR APRENDIZAJE	Menos importante
	**Proyecto final**<br>Implica considerable análisis y dedicación por parte del estudiante.<br>Las instrucciones son entregadas generalmente al inicio del semestre.<br>Tiene mucho peso en la calificación final.<br>Su realización es *individual o grupal*.	
	**Participación en clases**<br>Se considera la intervención del estudiante durante el desarrollo de la clase.<br>Su evaluación es continua durante el semestre.<br>Su evaluación es *individual*.	
	**Examen escrito supervisado**<br>Es de tiempo limitado y supervisado.<br>Se evalúa de forma remota mediante el uso de cámaras web para rastrear la actividad de los estudiantes durante el examen.<br>Su realización es *individual*.	
	**Evaluación de pares**<br>Es la retroalimentación dada por los compañeros basada en una serie de criterios tales como colaboración, esfuerzo en trabajos y actividades grupales.<br>Su evaluación puedes ser continua durante el semestre o al final del semestre.	

7. **Considerando solo estos 4 métodos de evaluación, indica:**
¿Cuál es el "Más importante" y cuál es el "Menos importante" para facilitar tu aprendizaje?

Más importante	MÉTODO DE EVALUACIÓN PARA FACILITAR APRENDIZAJE	Menos importante
	**Análisis y discusiones de casos reales**<br>Busca reflejar lo aprendido basado en análisis de casos de estudio.<br>El análisis se ve materializado en un documento escrito o exposición oral.<br>Para su evaluación se considera el dominio del tema, buena redacción y coherencia.<br>Su realización es *individual o grupal.*	
	**Presentaciones o demostraciones profesionales**<br>Se realizan sobre un tema en específico usando materiales audiovisuales.<br>Se evalúa el conocimiento de un tema y habilidades comunicativas.<br>Su realización es *individual o grupal.*	
	**Examen escrito supervisado**<br>Es de tiempo limitado y supervisado.<br>Se evalúa de forma remota mediante el uso de cámaras web para rastrear la actividad de los estudiantes durante el examen.<br>Su realización es *individual.*	
	**Talleres, laboratorios y simulaciones**<br>Son evaluaciones basadas en actividades prácticas.<br>Permite que alumnos ensayen habilidades técnicas.<br>Su realización es *individual o grupal.*	

8. **Considerando solo estos 4 métodos de evaluación, indica:**
¿Cuál es el "Más importante" y cuál es el "Menos importante" para facilitar tu aprendizaje?

Más importante	MÉTODO DE EVALUACIÓN PARA FACILITAR APRENDIZAJE	Menos importante
	**Análisis y discusiones de casos reales**<br>Busca reflejar lo aprendido basado en análisis de casos de estudio.<br>El análisis se ve materializado en un documento escrito o exposición oral.<br>Para su evaluación se considera el dominio del tema, buena redacción y coherencia.<br>Su realización es *individual o grupal.*	
	**Portafolio**<br>Es una compilación de trabajos que el estudiante entrega de forma recurrente.<br>Su evaluación puede ser continua durante el semestre o al final del semestre.<br>Puede incluir trabajos como: documentos escritos, trabajos manuales y registros audiovisuales.<br>Su realización es *individual.*	
	**Pruebas continuas de preguntas abiertas**<br>Pueden realizarse en modalidad escrita u oral.<br>Cada pregunta evalúa más de una habilidad o contenido.<br>Miden el conocimiento basados en las propias respuestas del estudiante.<br>Su realización es *individual*	
	**Evaluación de pares**<br>Es la retroalimentación dada por los compañeros basada en una serie de criterios tales como colaboración, esfuerzo en trabajos y actividades grupales.<br>Su evaluación puedes ser continua durante el semestre o al final del semestre.	

9. **Considerando solo estos 4 métodos de evaluación, indica:**
¿Cuál es el “Más importante" y cuál es el "Menos importante" para facilitar tu aprendizaje?

Más importante	MÉTODO DE EVALUACIÓN PARA FACILITAR APRENDIZAJE	Menos importante
	**Proyecto final**<br>Implica considerable análisis y dedicación por parte del estudiante.<br>Las instrucciones son entregadas generalmente al inicio del semestre.<br>Tiene mucho peso en la calificación final.<br>Su realización es *individual o grupal.*	
	**Pruebas continuas de preguntas abiertas**<br>Pueden realizarse en modalidad escrita u oral.<br>Cada pregunta evalúa más de una habilidad o contenido.<br>Miden el conocimiento basados en las propias respuestas del estudiante.<br>Su realización es *individual.*	
	**Exámenes de libro abierto**<br>Contienen preguntas de análisis que no se pueden encontrar fácilmente en ninguna fuente (internet y libros).<br>Mide el conocimiento profundo a través de la aplicación de lo aprendido.	
	**Talleres, laboratorios y simulaciones**<br>Son evaluaciones basadas en actividades prácticas.<br>Permite que alumnos ensayen habilidades técnicas.<br>Su realización es *individual o grupal.*	

10. **Considerando solo estos 4 métodos de evaluación, indica:**
¿Cuál es el “Más importante" y cuál es el "Menos importante" para facilitar tu aprendizaje?

Más importante	MÉTODO DE EVALUACIÓN PARA FACILITAR APRENDIZAJE	Menos importante
	**Proyecto final**<br>Implica considerable análisis y dedicación por parte del estudiante.<br>Las instrucciones son entregadas generalmente al inicio del semestre.<br>Tiene mucho peso en la calificación final.<br>Su realización es *individual o grupal.*	
	**Tareas en casa**<br>Se evalúa el trabajo realizado fuera del horario de clases.<br>Los plazos de entrega suelen ser cortos.<br>Su evaluación es continua durante el semestre.<br>Su realización es generalmente *individual.*	
	**Portafolio**<br>Es una compilación de trabajos que el estudiante entrega de forma recurrente.<br>Su evaluación puede ser continua durante el semestre o al final del semestre.<br>Puede incluir trabajos como: documentos escritos, trabajos manuales y registros audiovisuales.<br>Su realización es *individual.*	
	**Presentaciones o demostraciones profesionales**<br>Se realizan sobre un tema en específico usando materiales audiovisuales.<br>Se evalúa el conocimiento de un tema y habilidades comunicativas.<br>Su realización es *individual o grupal.*	

11. **Considerando solo estos 4 métodos de evaluación, indica:**
¿Cuál es el “Más importante" y cuál es el "Menos importante" para facilitar tu aprendizaje?

Más importante	MÉTODO DE EVALUACIÓN PARA FACILITAR APRENDIZAJE	Menos importante
	**Tareas en casa**<br>Se evalúa el trabajo realizado fuera del horario de clases.<br>Los plazos de entrega suelen ser cortos.<br>Su evaluación es continua durante el semestre.<br>Su realización es generalmente *individual.*	
	**Ensayo escrito**<br>Se evalúa el conocimiento sobre un tema específico y la opinión del estudiante.<br>Se evalúa el uso de la información (teoría) para emitir juicios.<br>Su realización es *individual.*	
	**Evaluación de pares**<br>Es la retroalimentación dada por los compañeros basada en una serie de criterios tales como colaboración, esfuerzo en trabajos y actividades grupales.<br>Su evaluación puedes ser continua durante el semestre o al final del semestre.	
	**Talleres, laboratorios y simulaciones**<br>Son evaluaciones basadas en actividades prácticas.<br>Permite que alumnos ensayen habilidades técnicas.<br>Su realización es *individual o grupal.*	

12. **Considerando solo estos 4 métodos de evaluación, indica:**
¿Cuál es el “Más importante" y cuál es el "Menos importante" para facilitar tu aprendizaje?

Más importante	MÉTODO DE EVALUACIÓN PARA FACILITAR APRENDIZAJE	Menos importante
	**Ensayo escrito**<br>Se evalúa el conocimiento sobre un tema específico y la opinión del estudiante.<br>Se evalúa el uso de la información (teoría) para emitir juicios.<br>Su realización es *individual.*	
	**Portafolio**<br>Es una compilación de trabajos que el estudiante entrega de forma recurrente.<br>Su evaluación puede ser continua durante el semestre o al final del semestre.<br>Puede incluir trabajos como: documentos escritos, trabajos manuales y registros audiovisuales.<br>Su realización es *individual.*	
	**Exámenes de libro abierto**<br>Contienen preguntas de análisis que no se pueden encontrar fácilmente en ninguna fuente (internet y libros).<br>Mide el conocimiento profundo a través de la aplicación de lo aprendido.	
	**Examen escrito supervisado**<br>Es de tiempo limitado y supervisado.<br>Se evalúa de forma remota mediante el uso de cámaras web para rastrear la actividad de los estudiantes durante el examen.<br>Su realización es *individual.*	

13. Considerando solo estos 4 métodos de evaluación, indica:
¿Cuál es el “Más importante" y cuál es el "Menos importante" para facilitar tu aprendizaje?

Más importante	MÉTODO DE EVALUACIÓN PARA FACILITAR APRENDIZAJE	Menos importante
	**Pruebas continuas de opción múltiple**<br>Constan de preguntas las cuales tienen un número limitado de opciones de respuestas.<br>Cada pregunta evalúa una única habilidad y/o contenido.<br>Su realización es *individual*.	
	**Exámenes de libro abierto**<br>Contienen preguntas de análisis que no se pueden encontrar fácilmente en ninguna fuente (internet y libros).<br>Mide el conocimiento profundo a través de la aplicación de lo aprendido.	
	**Presentaciones o demostraciones profesionales**<br>Se realizan sobre un tema en específico usando materiales audiovisuales.<br>Se evalúa el conocimiento de un tema y habilidades comunicativas.<br>Su realización es *individual o grupal*.	
	**Evaluación de pares**<br>Es la retroalimentación dada por los compañeros basada en una serie de criterios tales como colaboración, esfuerzo en trabajos y actividades grupales.<br>Su evaluación puedes ser continua durante el semestre o al final del semestre.	

Ahora queremos conocer tu opinión considerando todos las opciones

14. ¿Cuál de todos los 13 métodos de evaluación consideras que son "Muy importantes" para facilitar tu aprendizaje? Escoge todos los métodos de evaluación que son "Muy importantes".

Considero que los siguientes métodos de evaluación son "muy importantes" para mi aprendizaje:

MÉTODOS DE EVALUACIÓN	
**Proyecto final**<br>Implica considerable análisis y dedicación por parte del estudiante.<br>Las instrucciones son entregadas generalmente al inicio del semestre.<br>Tiene mucho peso en la calificación final.<br>Su realización es *individual o grupal.*	
**Participación en clases**<br>Se considera la intervención del estudiante durante el desarrollo de la clase.<br>Su evaluación es continua durante el semestre.<br>Su evaluación es *individual.*	
**Tareas en casa**<br>Se evalúa el trabajo realizado fuera del horario de clases.<br>Los plazos de entrega suelen ser cortos.<br>Su evaluación es continua durante el semestre.<br>Su realización es generalmente *individual.*	
**Análisis y discusiones de casos reales**<br>Busca reflejar lo aprendido basado en análisis de casos de estudio.<br>El análisis se ve materializado en un documento escrito o exposición oral.<br>Para su evaluación se considera el dominio del tema, buena redacción y coherencia.<br>Su realización es *individual o grupal.*	
**Ensayo escrito**<br>Se evalúa el conocimiento sobre un tema específico y la opinión del estudiante.<br>Se evalúa el uso de la información (teoría) para emitir juicios.<br>Su realización es *individual.*	
**Portafolio**<br>Es una compilación de trabajos que el estudiante entrega de forma recurrente.<br>Su evaluación puede ser continua durante el semestre o al final del semestre.<br>Puede incluir trabajos como: documentos escritos, trabajos manuales y registros audiovisuales.<br>Su realización es *individual.*	
**Pruebas continuas de opción múltiple**<br>Constan de preguntas las cuales tienen un número limitado de opciones de respuestas.<br>Cada pregunta evalúa una única habilidad y/o contenido.<br>Su realización es individual.	
**Pruebas continuas de preguntas abiertas**<br>Pueden realizarse en modalidad escrita u oral.<br>Cada pregunta evalúa más de una habilidad o contenido.<br>Miden el conocimiento basados en las propias respuestas del estudiante.<br>Su realización es *individual.*	
**Exámenes de libro abierto**<br>Contienen preguntas de análisis que no se pueden encontrar fácilmente en ninguna fuente (internet y libros).<br>Mide el conocimiento profundo a través de la aplicación de lo aprendido.	
**Presentaciones o demostraciones profesionales**<br>Se realizan sobre un tema en específico usando materiales audiovisuales.<br>Se evalúa el conocimiento de un tema y habilidades comunicativas.<br>Su realización es *individual o grupal.*	
**Examen escrito supervisado**<br>Es de tiempo limitado y supervisado.<br>Se evalúa de forma remota mediante el uso de cámaras web para rastrear la actividad de los estudiantes durante el examen.<br>Su realización es *individual.*	
**Evaluación de pares**<br>Es la retroalimentación dada por los compañeros basada en una serie de criterios tales como colaboración, esfuerzo en trabajos y actividades grupales.<br>Su evaluación puedes ser continua durante el semestre o al final del semestre.	
**Talleres, laboratorios y simulaciones**<br>Son evaluaciones basadas en actividades prácticas.<br>Permite que alumnos ensayen habilidades técnicas.<br>Su realización es *individual o grupal.*	

Considero que ninguno de los 13 métodos de evaluación son "Muy importantes" para mi aprendizaje	

Ahora queremos conocer tu preferencia considerando todos las opciones

15. **¿Cuál de todos los 13 métodos de evaluación son los que "te gustan mucho", independientemente si facilita tu aprendizaje o no? Escoge todos los métodos de evaluación que "te gustan mucho".**

Considero que los siguientes métodos de evaluación "me gustan mucho", independientemente si creo que facilitan mi aprendizaje o no:

MÉTODOS DE EVALUACIÓN	
**Proyecto final**<br>Implica considerable análisis y dedicación por parte del estudiante.<br>Las instrucciones son entregadas generalmente al inicio del semestre.<br>Tiene mucho peso en la calificación final.<br>Su realización es *individual o grupal.*	
**Participación en clases**<br>Se considera la intervención del estudiante durante el desarrollo de la clase.<br>Su evaluación es continua durante el semestre.<br>Su evaluación es *individual.*	
**Tareas en casa**<br>Se evalúa el trabajo realizado fuera del horario de clases.<br>Los plazos de entrega suelen ser cortos.<br>Su evaluación es continua durante el semestre.<br>Su realización es generalmente *individual.*	
**Análisis y discusiones de casos reales**<br>Busca reflejar lo aprendido basado en análisis de casos de estudio.<br>El análisis se ve materializado en un documento escrito o exposición oral.<br>Para su evaluación se considera el dominio del tema, buena redacción y coherencia.<br>Su realización es *individual o grupal.*	
**Ensayo escrito**<br>Se evalúa el conocimiento sobre un tema específico y la opinión del estudiante.<br>Se evalúa el uso de la información (teoría) para emitir juicios.<br>Su realización es *individual.*	
**Portafolio**<br>Es una compilación de trabajos que el estudiante entrega de forma recurrente.<br>Su evaluación puede ser continua durante el semestre o al final del semestre.<br>Puede incluir trabajos como: documentos escritos, trabajos manuales y registros audiovisuales.<br>Su realización es *individual.*	
**Pruebas continuas de opción múltiple**<br>Constan de preguntas las cuales tienen un número limitado de opciones de respuestas.<br>Cada pregunta evalúa una única habilidad y/o contenido.<br>Su realización es individual.	
**Pruebas continuas de preguntas abiertas**<br>Pueden realizarse en modalidad escrita u oral.<br>Cada pregunta evalúa más de una habilidad o contenido.<br>Miden el conocimiento basados en las propias respuestas del estudiante.<br>Su realización es *individual.*	
**Exámenes de libro abierto**<br>Contienen preguntas de análisis que no se pueden encontrar fácilmente en ninguna fuente (internet y libros).<br>Mide el conocimiento profundo a través de la aplicación de lo aprendido.	
**Presentaciones o demostraciones profesionales**<br>Se realizan sobre un tema en específico usando materiales audiovisuales.<br>Se evalúa el conocimiento de un tema y habilidades comunicativas.<br>Su realización es *individual o grupal.*	
**Examen escrito supervisado**<br>Es de tiempo limitado y supervisado.<br>Se evalúa de forma remota mediante el uso de cámaras web para rastrear la actividad de los estudiantes durante el examen.<br>Su realización es *individual.*	
**Evaluación de pares**	

Es la retroalimentación dada por los compañeros basada en una serie de criterios tales como colaboración, esfuerzo en trabajos y actividades grupales.<br>Su evaluación puedes ser continua durante el semestre o al final del semestre.	
**Talleres, laboratorios y simulaciones**<br>Son evaluaciones basadas en actividades prácticas.<br>Permite que alumnos ensayen habilidades técnicas.<br>Su realización es *individual o grupal.*	

Considero que ninguno de los 13 métodos de evaluación "me gustan mucho", independientemente si creo que facilita mi aprendizaje o no.	

Imagina que tomas un curso en cualquier área de ciencias aplicadas que implementa todos estos 13 métodos de evaluación.

16. **¿Qué calificación esperarías recibir en cada formato método de evaluación? (Use un puntaje entre 1 a 7).**

Ejemplo:

Proyecto final	6
Participación en clases	1
Tareas en casa	3

Juan espera obtener un 6 de nota con este tipo de método de evaluación.

Juan espera obtener un 1 de nota con este tipo de método de evaluación.

Juan espera obtener un 3 de nota con este tipo de método de evaluación.

MÉTODOS DE EVALUACIÓN	Calificación esperada (1-7)
**Proyecto final**<br>Implica considerable análisis y dedicación por parte del estudiante.<br>Las instrucciones son entregadas generalmente al inicio del semestre.<br>Tiene mucho peso en la calificación final.<br>Su realización es *individual o grupal.*	
**Participación en clases**<br>Se considera la intervención del estudiante durante el desarrollo de la clase.<br>Su evaluación es continua durante el semestre.<br>Su evaluación es *individual.*	
**Tareas en casa**<br>Se evalúa el trabajo realizado fuera del horario de clases.<br>Los plazos de entrega suelen ser cortos.<br>Su evaluación es continua durante el semestre.<br>Su realización es generalmente *individual.*	
**Análisis y discusiones de casos reales**<br>Busca reflejar lo aprendido basado en análisis de casos de estudio.<br>El análisis se ve materializado en un documento escrito o exposición oral.<br>Para su evaluación se considera el dominio del tema, buena redacción y coherencia.<br>Su realización es *individual o grupal.*	
**Ensayo escrito**<br>Se evalúa el conocimiento sobre un tema específico y la opinión del estudiante.<br>Se evalúa el uso de la información (teoría) para emitir juicios.<br>Su realización es *individual.*	
**Portafolio**<br>Es una compilación de trabajos que el estudiante entrega de forma recurrente.<br>Su evaluación puede ser continua durante el semestre o al final del semestre.<br>Puede incluir trabajos como: documentos escritos, trabajos manuales y registros audiovisuales.<br>Su realización es *individual.*	

**Pruebas continuas de opción múltiple**<br>Constan de preguntas las cuales tienen un número limitado de opciones de respuestas.<br>Cada pregunta evalúa una única habilidad y/o contenido.<br>Su realización es individual.	
**Pruebas continuas de preguntas abiertas**<br>Pueden realizarse en modalidad escrita u oral.<br>Cada pregunta evalúa más de una habilidad o contenido.<br>Miden el conocimiento basados en las propias respuestas del estudiante.<br>Su realización es *individual*.	
**Exámenes de libro abierto**<br>Contienen preguntas de análisis que no se pueden encontrar fácilmente en ninguna fuente (internet y libros).<br>Mide el conocimiento profundo a través de la aplicación de lo aprendido.	
**Presentaciones o demostraciones profesionales**<br>Se realizan sobre un tema en específico usando materiales audiovisuales.<br>Se evalúa el conocimiento de un tema y habilidades comunicativas.<br>Su realización es *individual o grupal*.	
**Examen escrito supervisado**<br>Es de tiempo limitado y supervisado.<br>Se evalúa de forma remota mediante el uso de cámaras web para rastrear la actividad de los estudiantes durante el examen.<br>Su realización es *individual*.	
**Evaluación de pares**<br>Es la retroalimentación dada por los compañeros basada en una serie de criterios tales como colaboración, esfuerzo en trabajos y actividades grupales.<br>Su evaluación puedes ser continua durante el semestre o al final del semestre.	
**Talleres, laboratorios y simulaciones**<br>Son evaluaciones basadas en actividades prácticas.<br>Permite que alumnos ensayen habilidades técnicas.<br>Su realización es *individual o grupal*.	

Estilos de Aprendizaje

Ahora, te describiremos algunas definiciones. Por favor lee cuidadosamente y escoge la opción con la que más te identificas.

Considerando las siguientes definiciones de **estilos de aprendizaje:**

Estilo de Aprendizaje	Descripción
**ESTILO A**	
	Busca experiencias nuevas, son de mente abierta, nada escépticos y realizan con entusiasmo tareas nuevas. *Características: animador, improvisador, arriesgado y espontáneo.*
**ESTILO B**	
	Antepone la reflexión a la acción, observa con detenimiento las distintas experiencias. *Características: ponderado, concienzudo, receptivo, analítico y exhaustivo.*

ESTILO C

Necesita integrar la experiencia en un marco teórico de referencia. Busca la racionalidad y la objetividad huyendo de lo subjetivo y lo ambiguo.
Características: metódico, lógico, objetivo, crítico y Estructurado.

ESTILO D Le gusta actuar rápidamente y con seguridad con aquellas ideas y proyectos que les atraen.
Características: experimentador, práctico, directo y eficaz.

---

17. **¿Con qué estilo de aprendizaje te identificas más?**
    ( ) Estilo A *Características: animador, improvisador, arriesgado y espontáneo.*
    ( ) Estilo B *Características: ponderado, concienzudo, receptivo, analítico y exhaustivo.*
    ( ) Estilo C *Características: metódico, lógico, objetivo, crítico y estructurado.*
    ( ) Estilo D *Características: experimentador, práctico, directo y eficaz.*

18. **¿Con qué estilo de aprendizaje te identificas menos?**
    ( ) Estilo A *Características: animador, improvisador, arriesgado y espontáneo.*
    ( ) Estilo B *Características: ponderado, concienzudo, receptivo, analítico y exhaustivo.*
    ( ) Estilo C *Características: metódico, lógico, objetivo, crítico y estructurado.*
    ( ) Estilo D *Características: experimentador, práctico, directo y eficaz.*

Rasgos de personalidad

Considerando las siguientes definiciones de **rasgos de personalidad:**

Rasgos de personalidad	Descripción
**TIPO A**	Refiere al ajuste emocional, la tendencia a experimentar emociones negativas, baja tolerancia a la frustración, así como a comportamientos impulsivos.<br>*Cualidades: ansiedad, hostilidad, depresión, timidez, impulsividad, y vulnerabilidad.*
**TIPO B**	Refiere a la cantidad y calidad de interacciones interpersonales. Se compone de sociabilidad, alto nivel de actividad, asertividad y búsqueda de sensaciones.<br>*Cualidades: afecto, asertividad, actividad, búsqueda de emociones y Emociones positivas.*

TIPO C Refiere a la búsqueda de experiencias nuevas, originalidad, tendencia a la imaginación y atrevimiento. *Cualidades: fantasía, estética, sentimientos, acciones, ideas y valores.*

TIPO D Refiere a una actitud cooperativa, empática y comprometida con otras personas. *Cualidades: franqueza, altruismo, modestia, confianza, actitud conciliadora y sensibilidad interpersonal.*

TIPO E Refleja el grado de organización, persistencia, control y conducta dirigida a metas. *Cualidades son competencia, orden, necesidad de logro, sentido del deber, deliberación, y autodisclipina.*

---

19. **¿Con qué tipo de personalidad te identificas más?**
    ( ) Tipo A *Cualidades: ansiedad, hostilidad, depresión, timidez, impulsividad, y vulnerabilidad.*
    ( ) Tipo B *Cualidades: afecto, asertividad, actividad, búsqueda de emociones y Emociones positivas.*
    ( ) Tipo C *Cualidades: fantasía, estética, sentimientos, acciones, ideas y valores.*
    ( ) Tipo D *Cualidades: franqueza, altruismo, modestia, confianza, actitud conciliadora y sensibilidad interpersonal.*

    ( ) Tipo E *Cualidades: competencia, orden, necesidad de logro, sentido del deber, deliberación, y autodisclipina.*

20. **¿Con qué tipo de personalidad te identificas menos?**
    ( ) Tipo A *Cualidades: ansiedad, hostilidad, depresión, timidez, impulsividad, y vulnerabilidad.*
    ( ) Tipo B *Cualidades: afecto, asertividad, actividad, búsqueda de emociones y Emociones positivas.*
    ( ) Tipo C *Cualidades: fantasía, estética, sentimientos, acciones, ideas y valores.*
    ( ) Tipo D *Cualidades: franqueza, altruismo, modestia, confianza, actitud conciliadora y sensibilidad interpersonal.*
    ( ) Tipo E *Cualidades: competencia, orden, necesidad de logro, sentido del deber, deliberación, y autodisclipina.*

Considerando el contexto de confinamiento actual:

21. **¿Sientes que tienes que estudiar más para obtener buenas calificaciones en tus evaluaciones realizadas en clases en línea ?**
    ( ) Si

( ) No

22. **Los métodos de evaluación usados en tus clases en línea ¿Perjudicaron o beneficiaron tus calificaciones académicas?**
( ) Perjudicaron mucho mis calificaciones académicas.
( ) Perjudicaron poco mis calificaciones académicas.
( ) Ni perjudicaron ni beneficiaron mis calificaciones académicas.
( ) Beneficiaron poco mis calificaciones académicas.
( ) Beneficiaron mucho mis calificaciones académicas.

23. **¿Qué factor ha afectado tu desempeño durante las evaluaciones en línea desde el inicio de la pandemia? (Seleccione todas las que corresponda).**
( ) Falta de interacción con el profesor.
( ) Escasa participación por parte de tus compañeros.
( ) Capacidad de atención limitada.
( ) Falta de recursos (tecnológicos, físicos, entre otros).
( ) Carente preparación por parte de los docentes para educación en línea.
( ) Otros. Detallar: ___
( ) No he presentado ningún problema.

24. **¿Consideras que tu preferencia por los métodos de evaluación han cambiado a raíz de tu experiencia con clases en línea?**
( ) Si, ahora prefiero evaluaciones en línea.
( ) Si, ahora prefiero más evaluaciones presenciales.
( ) Si, ahora prefiero un sistema de evaluación que combine evaluaciones presenciales y en línea.
( ) No, siempre he preferido evaluaciones en línea.
( ) No, siempre he preferido evaluaciones presenciales.
( ) No, siempre he preferido un sistema de evaluación que combine evaluaciones presenciales y en línea.

Gracias por llegar tan lejos! Casi has terminado, lo prometemos. Solo quedan pocas preguntas más sobre ti.

25. **¿Consideras que la educación en línea (incluyendo evaluaciones en línea) es más eficiente (optimiza recursos y tiempo) para lograr los objetivos de aprendizaje tanto como la educación presencial?**
( ) Es mucho más eficiente
( ) Es eficiente
( ) Ni eficiente ni deficiente
( ) Es deficiente
( ) Es muy deficiente

26. **¿Cómo consideras que es el acceso a internet en tu hogar para el desarrollo de sus evaluaciones académicas?**
( ) Muy Bueno
( ) Bueno
( ) Regular
( ) Malo
( ) Muy malo

27. **¿Tú o tu familia recibe ayuda financiera (diferente a cualquier apoyo financiero para tus estudios como becas o créditos) por parte del estado/gobierno?**
( ) Si
( ) No
( ) No sé

28. **¿Cuál es la carrera profesional que estás estudiando?**
(_________)

29. **¿En qué año académico te encuentras?**
( ) Primer año
( ) Segundo año
( ) Tercer año
( ) Cuarto año
( ) Quinto año
( ) Sexto año
( ) Séptimo año

30. **¿Te identificas con alguno de estos grupos? Puedes marcar más de una opción.**
( ) De primera generación (estudiantes que son primeros en sus familias en asistir a la universidad).
( ) Perteneciente a un hogar con bajos ingresos.
( ) Perteneciente a una etnia u pueblo originario (Mapuche, Aymara, Diaguita, entre otros).
( ) Estudiantes de bajo rendimiento académico.
( ) Proviene de una zona rural.
( ) LGTBIQ+ (lesbiana, gay, bisexual, transgénero, transexual, trasgenero, intersexual y otros).
( ) Estudiantes con algún tipo de discapacidad.
( ) Ninguno

31. **Durante el periodo de tus clases en línea (en pandemia) ¿Has sentido que perteneces a un aula inclusiva? (aula que integra el aprendizaje y participación de todos los alumnos pese a sus diferencias raciales, sexuales, culturales, género, entre otros).**
( ) Mucho
( ) Bastante
( ) Regular
( ) Poco
( ) Nada

32. **Considerando las clases presenciales que tenías antes de la pandemia, ¿Sientes que perteneciste a un aula inclusiva? (aula que integra el aprendizaje y participación de todos los alumnos pese a sus diferencias raciales, sexuales, culturales, género, entre otros).**
( ) Mucho
( ) Bastante
( ) Regular
( ) Poco
( ) Nada

33. **¿Qué edad tienes?**
(___) años

34. ¿Con qué género te identificas?

( ) Masculino
( ) Femenino
( ) Prefiero no decir
( ) Otro:___

35. ¿Cuántos días hay en una semana?

( ) 1
( ) 2
( ) 3
( ) 4
( ) 5
( ) 6
( ) 7

36. Ingresa tu correo para que entres en el sorteo de la gift card: ____________________________
