# Supplementary material for: How to assess? Student preferences for methods to assess experiential learning: A best-worst scaling approach
Source: PLoS One. 2022 Oct 27;17(10):e0276745. doi: 10.1371/journal.pone.0276745 (PMC9612489; doi:10.1371/journal.pone.0276745)
Supplement: S2 File — (DOCX) [file pone.0276745.s017.docx]

**Analytical models**

In the discrete choice model, we assume that the indirect utility of respondent *n* derived from the selected alternative in a BW question *t* is defined by a deterministic utility component plus a stochastic error term $\epsilon_{nit}$:

$U_{nit}=\beta_{it}+ \epsilon_{nit}$, (1)

where 𝛽 is the vector of estimated importance parameters of the best alternative *i* relative to some option normalized to zero to ensure model identification. We estimated a mixed logit model (MXL) for panel data as heterogeneity in student preferences for assessments is expected. We assumed that $\epsilon_{nit}$ is independent and identically distributed (iid) type I extreme value. Given the sequence of BW choices over the *T* BW questions (*T*=13 in this study), the unconditional probability that an individual $n$ selects alternative *i* as the best and alternative *j* as the worst from a choice set can be expressed as follows:

$P_{n}(\beta)= \int_{\beta} \prod_{t=1}^{T} \frac{\exp\left( \beta_{nit -} \beta_{njt} \right)}{\sum_{q}^{J} \sum_{p=1}^{J} \exp\left( \beta_{nqt -} \beta_{npt} \right)} f\left( \beta_{n}|\theta\right)d\beta_{n}$, (2)

where $f\left( \beta_{n}|\theta\right)$ is the density of the importance parameters and $\theta$ are the parameters of the distribution. The model parameters are estimated by simulated maximum likelihood estimation technique using 500 Halton draws (Train, 2009).

To identify possible sources of heterogeneity, we estimated a latent class conditional logistic (LCL) model. This model creates *C* segments or classes of students with similar taste parameters $\beta$ and characteristics to account for differences in preferences (Hess et al., 2008). If a respondent *n* is in class *c*, the probability of observing this sequence of choices is given by the joint likelihood of respondent’s choices, given by

$P_{n}\left( \beta_{c} \right)=\prod_{t=1}^{T} \frac{\exp\left( \beta_{cnit -} \beta_{cnjt} \right)}{\sum_{q}^{J} \sum_{p=1}^{J} \exp\left( \beta_{cnqt -} \beta_{cnpt} \right)}$ (3)

The unconditional likelihood of agent $n$’s choices equals the weighted average of equation (3) over *C* classes. The weight for class $c$, $\pi_{cn}\left( \Theta\right),$is the share of the class modeled as fractional multinomial logit:

$\Pi_{cn}\left( \Theta\right)=\frac{\exp\left( z_{n}\theta_{c} \right)}{1+\sum_{m=1}^{C-1} \exp\left( z_{n}\theta_{m} \right)}$ (4)

where $\theta$ are class membership parameters in the model with $\theta^{C}$ normalized to zero for identification purposes and $z_{n}$ is a row vector of decision maker n’s characteristics with the standard constant regressor (that is 1). The joint likelihood of respondent’s $n$ responses is a function of the frequency of each class $C$, $\pi_{cn}\left( \Theta\right)$, and the class-specific utility parameters $\beta_{cnjt}$: $L_{n}(B,\Theta)=\sum_{c=1}^{C} \pi_{cn}(\Theta)P_{n}(\beta_{c})$.

We jointly estimate class membership and choice preferences as a function of individual characteristics in a LCL model using the expectation maximization (EM) algorithm (Pacifico & Yoo, 2013; Yoo, 2020). Both the MLX model and LCL model were estimated on the data exploded for our maxdiff decision process model using STATA (17.0).
